# Antihypertensive treatment and adverse psychiatric reactions in primary health care

**DOI:** 10.1038/s41371-026-01212-y

**Published:** 2026-08-27

**Authors:** Emina Curic, Henrik Ohlsson, Kenneth Kendler, Jan Sundquist, Kristina Sundquist, Margareta Reis, Bengt Zöller

**Affiliations:** 1 Center for Primary Health Care Research, Department of Clinical Sciences Malmö, Lund University, Malmö, Sweden; 2 University Clinic Primary Care, Skåne University Hospital, Region Skåne, Lund, Sweden; 3 Department of Clinical Pharmacology, Skåne University Hospital, Lund, Sweden; 4 Virginia Institute for Psychiatric and Behavioral Genetics, Virginia Commonwealth University, Richmond, VA USA; 5 Department of Biomedical and Clinical Sciences, Linköping University, Linköping, Sweden

**Keywords:** Hypertension, Risk factors

## Abstract

Antihypertensive treatment (AHT) has been associated with adverse psychiatric reactions (APRs), but existing data are conflicting. The aim of the present study was to determine the risk for seven different APRs in drug-naive patients with newly diagnosed hypertension in primary health care. This cohort study analyzed registry data of individuals aged 40–70 with a newly diagnosed untreated hypertension without previous psychiatric or cardiovascular comorbidities in Swedish primary healthcare from 2006 and onwards. Adjusted Cox proportional hazard ratios (aHRs) with 95% confidence intervals (CIs) were estimated with seven different AHTs as time-dependent variables. Seven different APRs outcomes were studied. Bonferroni correction was used. Among 677,564 studied individuals (51% female, mean age [±standard deviation] 58.0 [ ± 7.7] years), the highest risks of APRs occurred within the first two years of AHT. All AHTs were associated with one or more APRs. Beta-blockers showed strongest associations with APR. During the first two years after diagnosis of hypertension, beta-blockers were significantly associated with substance abuse disorders (aHR=1.48, 95%CI 1.43-1.54), psychotic disorders (aHR=1.46, 95%CI 1.27-1.68), affective disorders (aHR=1.35, 95%CI 1.32-1.39), bipolar disorders (aHR=1.34, 95%CI 1.12-1.61), depressive disorders (aHR=1.36, 95%CI 1.32-1.40), anxiety disorders (aHR=1.51, 95%CI 1.48-1.54), and sleep disorders (aHR=1.27, 95%CI 1.24-1.30). Certain associations remained in a co-sibling analysis. AHTs are associated with several APRs especially during the first two years after diagnosis of hypertension. However, it is important to note that only association but not causality can be inferred due to persistent confounding that could not be practically adjusted for.

## Introduction

Hypertension is one of the most common and important cardiovascular risk factors [1]. The prevalence of hypertension among adults in Sweden is estimated to be between 20 and 30% and more than 50% for seniors [2]. The prevalence of hypertension among adults aged 30-79 years in Europe is ranging from about 20% to 50% [3]. During 2017-2018 the prevalence of hypertension in USA was 54,5% aged 40-59 and 74,5% among the population aged 60 and over [4]. Antihypertensive treatment (AHT) drugs are commonly used to treat high blood pressure [1].

Depression frequently occurs among individuals with hypertension and various cardiovascular and cerebrovascular conditions [5]. The relationship between depression and cardiovascular disease is complex and a clear causal link has not yet been established [6]. Different mechanisms have been suggested, including hypothalamic–pituitary–adrenal (HPA) axis activation as a possible factor, which alters cardiovascular structure and function, and the sympathetic-adreno-medullary (SAM) axis [6]. Moreover, patients with depression often show increased sympathetic tone and reduced parasympathetic tone, lowering heart rate variability, and baroreflex sensitivity [6]. The prevalence of major depression in conditions such as hypertension, post-myocardial infarction, and post-stroke is around 30% depending on settings and method of evaluation [5].

An association between depression and hypertension is well established [7–9]. A meta-analysis found depression among 26,8% of hypertensive patients [7]. Another meta-analysis yielded similar results, with a prevalence of 29% for depression among older hypertensive patients [8]. Dysregulation in Ca^2+^ signaling has been implicated in the pathogenesis of both hypertension and depression, thus suggesting a possible contributary possibility between depression and hypertension [9]. However, depression and other psychiatric disorders may also be related to Anti-Hypertensive Treatment (AHT) with drugs [10–12]. The results regarding antihypertensive drugs and their correlation to psychiatric disorders are, however, conflicting. A Norwegian study found no association with AHT and depression or anxiety in patients who received a single drug AHT [12] A systematic review concluded that beta-blockers (BBs) were associated with sleep disorders but not with depression [13]. Moreover, a large cohort study in Sweden could not link prescriptions of BBs and hospital treated psychiatric disorders [14].

An American cohort study of electronic health records indicated that Angiotensin Receptor Blockers (ARBs) were the most advantageous drug regarding the risk for psychiatric disorders, while BBs were the least advantageous [11]. Thus, different classes of anti-hypertensive classes, i.e. Calcium Channel Blockers (CCBs), diuretics, Angiotensin-Converting Enzyme Inhibitors (ACEIs), ARBs, and BBs, may have different Adverse Psychiatric Reactions (APRs) [5, 11].

The present study is the first large primary care-based registry study to determine the association between seven anti-hypertensive drug classes and adverse psychiatric reactions. This Swedish primary healthcare study includes drug-naive patients with hypertension, without previous mental disorders or cardiovascular disease other than hypertension. Moreover, in a co-sibling design using siblings discordant for the exposure, adjustments were made for familial resemblance [15].

## Materials and methods

### Data sources

Data were obtained by linking several Swedish national population-based registers using the unique personal identity number assigned to all Swedish residents [16]. This identifier enables accurate linkage across datasets. To ensure confidentiality, Statistics Sweden (www.scb.se) replaced each personal identity number with a pseudonymized serial number before data delivery [16, 17].

The study used several Swedish national registers with well-documented completeness and validity [17]. We also used primary care data collected from almost all regions in Sweden. The primary care data includes records from 20 of 21 Swedish regions, covering approximately 87% of the population, with data available from the year 2000 onward [18, 19]. The National Patient Register (NPR) contains nationwide inpatient care data since 1987 and specialist outpatient care data since 2001 [20–22]. The Swedish Prescribed Drug Register provides comprehensive information on all dispensed medications in Sweden from July 2005 and onwards [23]. The Multi-Generation Register offers familial linkage data for individuals born in 1932 or later who have resided in Sweden since 1961 [20, 24]. The nationwide register data were accessed through Statistics Sweden and the Swedish National Board of Health and Welfare (www.socialstyrelsen.se).

### Study population and design

This was a historic cohort register-based study. The included individuals were aged 40-70 years and registered with essential (primary) hypertension ICD-10 (International Classification of Disease Code) I10 in the Swedish primary care data that were used in this study from 1st January 2006 until 31st December 2018. Individuals below the age of 40 were not included to decrease the possibility of secondary hypertension [25]. Older individuals than 70 years were not included due to an increased risk for multimorbidity [26].

Hypertensive primary care patients with previous prescriptions of AHT (CCBs, diuretics, ACE inhibitors, ARBs, BBs, imidazole receptor agonists, or alpha adrenoreceptor agonists) from 31st July 2005 to 31st December 2005 were excluded. Patients with a previous psychiatric disorder in the NPR or the primary care data were excluded to ensure detection of only incident cases of APRs as study outcomes. Pharmacoepidemiologic studies of AHTs can be misperceived by confounding by indication [11]. Patients were therefore also excluded if they had a previous diagnosis in the NPR or the primary care data of coronary heart disease, atrial fibrillation, heart failure, cerebrovascular disease, and prostate disease (often treated with alpha-adrenoreceptor antagonists). Details of the ICD-10 codes used are listed in Table S1. The total number of hypertensive patients included in the study after exclusions was 677,564.

### Main outcome variables

The outcomes in the study were the following seven psychiatric disorders: 1) substance abuse disorders, 2) psychotic disorders, 3) affective disorders, 4) bipolar disorders, 5) depressive disorders, 6) anxiety disorders, and 7) sleep disorders. Details of the ICD-10 codes are listed in Table S2. The outcome variables for APRs registered were obtained from primary health care data and NPR. Bipolar and depressive disorders are included in the group with affective disorders, but analyses are made in separate models.

### Main predictors

From the Swedish drug register, a total of 18 ATC (Anatomical Therapeutic Chemical Classification System) codes were included with the major approved antihypertensive drug classes in Sweden. Those antihypertensive drugs were the main predictors and included the following antihypertensive drugs: CCBs, diuretics, ACE inhibitors, ARBs, BBs, imidazoline receptor agonists, or alpha-adrenoreceptor antagonists. Details of the ATC codes are listed in Table S3. Since BBs differ in lipophilicity and central nervous system penetration—factors that may mask drug-specific associations [27, 28]—we performed an additional analysis of the seven beta-blockers used in Sweden: metoprolol, atenolol, bisoprolol, propranolol, sotalol, labetalol, and carvedilol. Propranolol is highly lipophilic, metoprolol bisoprolol, and carvedilol are moderate lipophilic, and atenolol, sotalol, and labetalol are low lipophilic or hydrophilic beta-blockers [27, 28].

### Adjusting variables

Adjustments were made for year of birth, age at onset of hypertension, sex, level of education, type of neighborhood [29], and country of birth (Tables S4 and S5). Adjustments were made for diabetes mellitus and thyroid disorders from primary health care data and NPR before baseline, i.e. diagnosis of hypertension in primary care. There is a range of factors that may influence the risk of potential APRs [11]. Adjustments were therefore also made for prescriptions of the following drugs, which had been dispensed prior to the recorded diagnosis of hypertension i.e. before baseline: opioids, neuroleptics, anxiolytics and sleep aids, antidepressants, metformin, and statins (Table S4).

### Statistical analysis

Survival analysis using Cox proportional hazards regression was used to investigate the association between anti-hypertensive drugs and psychiatric disorders, with AHT as a time-dependent variable comparing exposed with unexposed individuals for respective AHT. AHT was used as time-dependent variables because switching is common in hypertension treatment. Time-dependent exposure was determined using an intention to treat principle, patients once exposed were counted as exposed even if the specific AHT therapy was discontinued. Comparison groups are those unexposed to respective AHT class. Results are presented as adjusted Hazard Ratios (aHRs) with 95% confidence intervals (CIs). Treatment duration time with the drugs was analyzed separately either by treatment 0-2 years or two years and longer from time of hypertension diagnosis. The arbitrary stratification of years was chosen to permit stabilization of blood pressure responses to therapy and to capture the full spectrum of expected adverse reactions (Table S6) [30, 31]. This is also a pragmatic way to test for possible violations against proportional hazards assumption. Stratified analysis was performed according to men and women. Cox regression models were made from first diagnosis of hypertension in the primary care data until time to outcome (i.e. psychiatric disorder), death, loss of follow-up, or end of follow-up. All outcome variables were analyzed in separate models and were not mutually exclusive. Three different models were analyzed: Model A, B, and C. Each of the hypertensive drugs was investigated in separate models A and B. Model A was adjusted for year of birth, age at hypertension onset, education, neighborhood, and country of birth. Model B was adjusted for variables adjusted for in Model A but also for baseline diagnosis of diabetes mellitus, thyroid disorders, opioids, neuroleptics, anxiolytics and sleep aids, antidepressants, metformin, and statins. The fully adjusted Model C included the same covariates as Model B. In addition, all the studies antihypertensive agents were incorporated into a single multivariable model because combination therapy is common (Table S7). Moreover, we conducted a co-relative analysis of hypertensive siblings discordant for exposure, i.e. anti-hypertensive drug treatment [15]. In cases where there were more than two siblings, we performed repeated matching, pairing sibling 1 with sibling 2, sibling 1 with sibling 3, and sibling 2 with sibling 3. Statistical analysis was performed using SAS version 9.3 (SAS Institute Inc) and STATA. We calculated a Bonferroni (0.05/(7x7x2)) corrected significance threshold of *P* < 0.00051, which was considered significant. Bonferroni correction was based on the testing of seven different anti-hypertensive drugs and seven different outcomes analyzed for two different follow-up periods (Table S8). Bonferroni correction was used to reduce the risk of false positives since conducting multiple comparisons was achieved by adjusting the significance threshold.

### Ethics

Individual informed consent was not obtained, as this study used secondary, pseudonymized nationwide register data. The present study was a non-intervention register study based on encrypted secondary data obtained from the Swedish authorities and was approved by the Ethical Review Board in Lund, Sweden, 2013-02-15 (Dnr. 2012/795 and later approved amendments).

## Results

### Descriptive data

The total number of individuals included in the study was 677,564 (Table 1), 51% (*N* = 346,842) of these were females. The mean age of hypertension onset was 58 years. Most of the population had a middle level of education (47.6%) while 23.3% of the population had low education and 28.2% had high education (Table S5). Most of the study population were Swedish-born with Swedish-born parents (76.8%). The prevalence of prescribed antidepressive drugs was twice as high in females (10.1%) compared to males (5.0%). A similar pattern was observed in the prescription of anxiolytics and sleep aids, with a prevalence of 16.7% for females and 9.9% for males. Prescription and use of imidazoline receptor agonists and alpha-adrenoreceptor antagonists were very low (0.31%–2.09%) in the study population compared to other included antihypertensive drugs (43.3% –55.6%). For every outcome, there is a different follow-up time, and the minimum and maximum calculated use of respective AHT drug classes are given.

**Table 1:** Descriptive table about the study population.

	All	Female	Male
Total number of included individuals.	677,564	346,842	330,722
*Adjusting variables*			
Age at hypertension, mean (SD), years	58.0 (7.7)	58.6 (7.7)	57.2 (7.8)
Year of birth (SD)	1952 (9.2)	1951 (9.1)	1953 (9.2)
**Education**			
Missing	6,647 (1.0%)	3,851 (1.1%)	2,796 (0.9%)
Low	157,606 (23.3%)	77,174 (22.3%)	80,432 (24.3%)
Mid	322,218 (47,6%)	163,669 (47.2%)	158,549 (47.9%)
High	191,093 (28.2%)	102,148 (29.5%)	88,945 (26.9%)
**Type of neighborhood**			
Low Deprivation	142,935 (21.1%)	73,238 (21.1%)	69,697 (21.1%)
Mid Deprivation	423,828 (62.6%)	215,460 (62.1%)	208,368 (63.0%)
High Deprivation	110,801 (16.4%)	58,144 (16.8%)	52,657 (15.9%)
Country of birth			
Native-born – Native-born parents	520,133 (76.8%)	264,790 (76.3%)	255,343 (77.2%)
Native-born – One parent born outside Sweden	30,421 (4.5%)	14,390 (4.2%)	16,031 (4.9%)
Native-born – Two parents born outside Sweden	11,124 (1.6%)	5,186 (1.5%)	5,938 (1.8%)
Born outside Sweden	115,886 (17.1%)	62,476 (18.0%)	53,410 (16.2%)
Diabetes mellitus	88,695 (13.1%)	36,384 (10.5%)	52,311 (15.8%)
Thyroid disorders	41,158 (6.1%)	34,674 (10.0%)	6,484 (2.0%)
Opioids	160,470 (23.7%)	85,221 (24.6%)	75,249 (22.8%)
Neuroleptics	2,850 (0.4%)	1,745 (0.5%)	1,105 (0.3%)
Anxiolytics and sleep aids	90,654 (13.4%)	57,956 (16.7%)	32,698 (9.9%)
Antidepressive	51,369 (7.6%)	57,593 (10.1%)	16,505 (5.0%)
Metformin	44,981 (6.6%)	17,714 (5.1%)	27,267 (8.2%)
Statins	123,476 (18.2%)	57,593 (16.6%)	65,883 (19.9%)
Main Predictors*			
Calcium channel blocker*	46.2% - 48.5%	43.0% - 46.2%	49.4% - 50.9%
Diuretics*	50.6% - 52.2%	53.8% - 55.9%	47.2% - 48.3%
Angiotensin-Converting Enzyme inhibitors (ACE inhibitors)*	54.4% - 55.6%	50.9% - 52.7%	58.0% - 58.7%
Angiotensin II Receptor Blockers (ARB)*	43.9% - 45.7%	43.8% - 46.3%	44.0% - 45.1%
Beta blockers (BB)*	43.3% - 44.9%	46.7% - 48.8%	39.7% - 40.8%
Imidazoline receptor agonists*	0.31% - 0.35%	0.29% - 0.33%	0.35% - 0.37%
Alpha-adrenoreceptor antagonists*	1.90% - 2.09%	1.22% - 1.39%	2.67% - 2.84%
*Outcomes*			
Substance abuse disorders (ICD10 F10-F19)	5.7%	4.8%	6.5%
Psychotic disorders (ICD10 F20-F29)	0.34%	0.39%	0.28%
Affective disorders (ICD10 F30-F39)	9.0%	11.3%	6.5%
Bipolar disorders (ICD10 F30, F31)	0.20%	0.22%	0.18%
Depressive disorders(ICD10 F32, F33)	8.7%	11.0%	6.3%
Anxiety disorders (ICD10 F40-F48)	13.8%	18.0%	9.2%
Sleep disorders (ICD10 F51, G47)	14.0%	15.6%	12.3%

*For every outcome there is different follow-up time and the minimum and maximum calculated use of respective antihypertensive (AHT) drug class are given.

*SD* standard deviation.

### Association between AHT and psychiatric disorders

There was no significant difference between sex and all outcomes (data not shown in tables). Stratified analyses for sex are presented in Table S9 and Table S10. Results for both sexes are presented together in the main analysis (Table 2 and Table S11). Stratified analyses for sex are presented in Table S9 and Table S10. Generally, the risk for developing psychiatric disorders was highest during the two first years of follow-up after a diagnosis of hypertension and decreased after two years (Table 2). There were generally only minor differences between models A and B (Table S11). However, in the fully adjusted model C, with all anti-hypertensive drugs included in the same model, the aHRs were generally considerably lower than in models A and B (Table S11). The strongest association was observed for BBs that was significant in model C for all conditions except bipolar disorders (Table 2). Below are the individual psychiatric outcomes presented.

**Table 2:** Cox regression model C with antihypertensive treatment as a time dependent variable.

MALES AND FEMALES		MODEL C	
		0-2 years	2 + years
OUTCOME	PREDICTOR	HR 95% CI	HR 95% CI
Substance abuse disorders	Calcium channel blocker (CCB)	1.34 (1.30; 1.38)*	1.15 (1.12; 1.18)*
	Diuretics	1.13 (1.09; 1.17)*	1.10 (1.08; 1.13)*
	Angiotensin-Converting Enzyme inhibitors (ACE inhibitors)	1.23 (1.19; 1.27)*	1.05 (1.03; 1.08)*
	Angiotensin II Receptor Blockers (ARB)	1.04 (1.00; 1.08)	0.92 (0.89; 0.94)*
	Beta blockers (BB)	1.48 (1.43; 1.54)*	1.23 (1.20; 1.26)*
	Imidazoline receptor agonists	1.48 (1.10; 1.99)	0.98 (0.77; 1.24)
	Alpha-adrenoreceptor antagonists	0.72 (0.61; 0.84)*	0.74 (0.65; 0.83)*
Psychotic disorders	Calcium channel blocker (CCB)	1.04 (0.91; 1.20)	1.00 (0.90; 1.12)
	Diuretics	1.00 (0.86; 1.15)	0.94 (0.85; 1.04)
	Angiotensin-Converting Enzyme inhibitors (ACE inhibitors)	0.92 (0.80; 1.07)	0.92 (0.84; 1.02)
	Angiotensin II Receptor Blockers (ARB)	0.76 (0.64; 0.90)	0.82 (0.73; 0.91)*
	Beta blockers (BB)	1.46 (1.27; 1.68)*	1.17 (1.06; 1.29)
	Imidazoline receptor agonists	1.07 (0.26; 4.43)	1.13 (0.46; 2.75)
	Alpha-adrenoreceptor antagonists	1.51 (0.90; 2.54)	0.70 (0.41; 1.19)
Affective disorders	Calcium channel blocker (CCB)	1.17 (1.14; 1.20)*	1.05 (1.02; 1.07)*
	Diuretics	1.09 (1.06; 1.12)*	1.06 (1.04; 1.08)*
	Angiotensin-Converting Enzyme inhibitors (ACE inhibitors)	1.04 (1.02; 1.07)	0.99 (0.97; 1.01)
	Angiotensin II Receptor Blockers (ARB)	1.10 (1.07; 1.13)*	1.04 (1.02; 1.06)*
	Beta blockers (BB)	1.35 (1.32; 1.39)*	1.09 (1.07; 1.11)*
	Imidazoline receptor agonists	1.16 (0.89; 1.50)	1.12 (0.94; 1.32)
	Alpha-adrenoreceptor antagonists	1.16 (1.03; 1.30)	1.08 (0.98; 1.18)
Bipolar disorders	Calcium channel blocker (CCB)	0.90 (0.74; 1.09)	0.92 (0.80; 1.07)
	Diuretics	1.18 (0.98; 1.41)	1.04 (0.91; 1.18)
	Angiotensin-Converting Enzyme inhibitors (ACE inhibitors)	0.94 (0.78; 1.14)	0.82 (0.72; 0.94)
	Angiotensin II Receptor Blockers (ARB)	0.93 (0.76; 1.13)	0.93 (0.80; 1.07)
	Beta blockers (BB)	1.34 (1.12; 1.61)	1.05 (0.92; 1.19)
	Imidazoline receptor agonists	0.98 (0.14; 6.63)	1.04 (0.33; 3.25)
	Alpha-adrenoreceptor antagonists	0.71 (0.27; 1.86)	1.06 (0.58; 1.93)
Depressive disorders	Calcium channel blocker (CCB)	1.18 (1.14; 1.21)*	1.04 (1.02; 1.07)*
	Diuretics	1.09 (1.06; 1.12)*	1.07 (1.05; 1.09)*
	Angiotensin-Converting Enzyme inhibitors (ACE inhibitors)	1.05 (1.02; 1.08)	0.99 (0.97; 1.01)
	Angiotensin II Receptor Blockers (ARB)	1.10 (1.07; 1.14)*	1.04 (1.02; 1.06)*
	Beta blockers (BB)	1.36 (1.32; 1.40)*	1.10 (1.08; 1.12)*
	Imidazoline receptor agonists	1.19 (0.92; 1.54)	1.12 (0.95; 1.33)
	Alpha-adrenoreceptor antagonists	1.18 (1.05; 1.32)	1.07 (0.98; 1.17)
Anxiety disorders	Calcium channel blocker (CCB)	1.21 (1.19; 1.24)*	1.02 (1.00; 1.03)*
	Diuretics	1.00 (0.98; 1.03)*	0.99 (0.97; 1.00)*
	Angiotensin-Converting Enzyme inhibitors (ACE inhibitors)	1.07 (1.04; 1.09)	0.97 (0.95; 0.98)
	Angiotensin II Receptor Blockers (ARB)	1.15 (1.13; 1.18)*	1.06 (1.04; 1.08)*
	Beta blockers (BB)	1.51 (1.48; 1.54)*	1.13 (1.12; 1.15)*
	Imidazoline receptor agonists	1.22 (0.98; 1.51)	1.27 (1.10; 1.46)
	Alpha-adrenoreceptor antagonists	1.08 (0.98; 1.19)	0.94 (0.87; 1.02)
Sleep disorders	Calcium channel blocker (CCB)	1.25 (1.23; 1.28)*	1.06 (1.04; 1.08)*
	Diuretics	1.12 (1.10; 1.15)*	1.12 (1.10; 1.14)*
	Angiotensin-Converting Enzyme inhibitors (ACE inhibitors)	1.14 (1.12; 1.17)*	0.98 (0.97; 1.00)
	Angiotensin II Receptor Blockers (ARB)	1.24 (1.21; 1.27)*	1.14 (1.12; 1.15)*
	Beta blockers (BB)	1.27 (1.24; 1.30)*	1.12 (1.10; 1.13)*
	Imidazoline receptor agonists	1.54 (1.29; 1.85)*	1.05 (0.92; 1.21)
	Alpha-adrenoreceptor antagonists	1.23 (1.13; 1.34)*	1.00 (0.94; 1.07)

Model C: Adjusted for Year of birth, Age at Hypertension, Education, Type of neighborhood, Country of birth, Diabetes mellitus, Thyroid disorders, Opioids, Neuroleptics, Anxiolytics and sleep aids, Antidepressive, Metformin, and Statins. All hypertensive drugs were included in the same model.

Significant associations after Bonferroni correction are marked with an asterisk (*).

### Substance abuse disorders

In the fully adjusted Model C, after Bonferroni correction (Table 2), the risk of substance abuse disorders was significantly increased within the first two years after hypertension diagnosis for treatment with CCBs (aHR=1.34, 95% CI 1.30-1.38), diuretics (aHR= 1.13, 95% CI 1.09-1.17), ACEIs (aHR=1.23, 95% CI 1.19-1.27), and the highest risk for BBs (aHR=1.48, 95% CI 1.43-1.54). Treatment with alpha-adrenoreceptor antagonists was associated with a lower risk of substance abuse disorders during the first two years after hypertension diagnosis (aHR= 0,72, 95% CI 0,61-0,84).

### Psychotic disorders

Patients who were treated with BBs, for 0-2 years of follow-up, had a significant association with increased risk (aHR=1.46, 95% CI 1.27-1.68) for psychotic disorders. Patients who were treated with ARB, for at least two years after hypertension diagnosis, had a significantly decreased risk (aHR=0.82, 95% CI 0.73-0.91) to develop psychotic disorders (Table 2).

### Affective disorders

There was a significantly increased association for affective disorders, during the two first years of follow-up, with CCBs (aHR=1.17, 95% CI 1.14-1.20) diuretics (aHR=1.09, 95% CI 1.06-1.12), ARBs (aHR=1.10, 95% CI 1.07-1.13), and BBs (aHR=1.35, 95% CI 1.32-1.39). After two years of follow-up, the significantly increased association for affective disorders remained for these four drugs (Table 2).

### Bipolar disorders

During the first two years of follow-up no significant associations with bipolar disorders were observed after Bonferroni correction.

### Depressive disorders

There was a significantly increased association for depressive disorders, during the first two years of follow-up, with CCBs (HR = 1.18, 95% CI 1.14-1.21), diuretics (HR = 1.09, 95% CI 1.06-1.12), ARBs (HR = 1.10, 95% CI 1.07-1.14), and BBs (HR = 1.36, 95% CI 1.32-1.40) (Model C in Table 2). After two years of treatment, the significantly increased association for depressive disorders remained for these four drugs.

### Anxiety disorders

There was a significantly increased association for anxiety disorders, during the two first years of follow-up, with CCBs (HR = 1.21, 95% CI 1.19-1.24), ARBs (HR = 1.15 95% CI 1.13; 1.18), and BBs (HR = 1.51, 95% CI 1.48-1.54) (Table 2). After two years of follow-up, the significantly increased association for affective disorders remained for ARBs and BBs.

### Sleep disorders

There was a significantly increased association for sleep disorders while being followed for 0-2 years for all seven classes of anti-hypertensive drugs. After two or more years of follow-up, a significantly increased risk for sleep disorders remained for CCBs, diuretics, ARBs, and BBs (Table 2).

### Associations between individual beta-blockers and psychiatric disorder outcomes (Table 3)

Generally, for the individual beta-blockers the HR for developing psychiatric disorders was highest during the first two years of follow-up after a diagnosis of hypertension and decreased after two years (Table 3). The low lipophilic beta-blocker atenolol had no or only slightly increased HR for developing psychiatric disorders. The highly lipophilic betablocker propranolol had generally the highest HRs for developing psychiatric disorders especially during the first two years of follow-up. For instance, the HR for developing anxiety disorders for propranolol users was 2.29 (95% CI 2.13-2.46) during the first two years of follow-up. In comparison, the moderate lipophilic beta-blocker metoprolol had HR of 1.48 (95% CI 1.44-1.52) for developing anxiety disorders during the first two years of follow-up while the low lipophilic beta-blocker atenolol had HR of 1.19 (95% CI 1.14-1.24). However, there were exceptions from the rule that lipophilic beta-blockers are associated with more psychiatric adverse reactions [27, 28]. The low lipophilic betablocker sotalol had a HR of 1.87 (95% CI 1.71-2.05) for developing depressive disorders the first two years of follow-up.

**Table 3:** Cox regression model C with individual beta blockers treatment as a time dependent variable.

MALES AND FEMALES		MODEL C	
		0-2 years	2 + years
OUTCOME	PREDICTOR	HR 95% CI	HR 95% CI
Substance abuse disorders	Metoprolol (moderate)	1.54 (1.47; 1.61)	1.13 (1.10; 1.17)
	Atenolol (low)	1.00 (0.93; 1.08)	1.13 (1.10; 1.17)
	Bisoprolol (moderate)	1.63 (1.49; 1.79)	1.08 (1.02; 1.15)
	Propranolol (high)	1.84 (1.62; 2.10)	1.09 (1.02; 1.17)
	Sotalol (Low)	0.72 (0.44; 1.15)	0.88 (0.72; 1.07)
	Labetalol (Low)	1.27 (0.68; 2.35)	0.64 (0.45; 0.90)
	Carvedilol (Moderate)	0.89 (0.65; 1.24)	0.99 (0.86, 1.13)
Psychotic disorders	Metoprolol (moderate)	1.47 (1.24; 1.74)	1.15 (1.04; 1.29)
	Atenolol (low)	1.35 (1.06; 1.72)	0.96 (0.85; 1.09)
	Bisoprolol (moderate)	1.55 (1.08; 2.22)	1.37 (1.10; 1.70)
	Propranolol (high)	1.49 (0.90; 2.48)	1.27 (1.00; 1.62)
	Sotalol (Low)	0.53 (0.07; 3.74)	1.22 (0.67; 2.22)
	Labetalol (Low)	2.27 (0.32; 16.0)	0.87 (0.22; 3.49)
	Carvedilol (Moderate)	0.63 (0.16; 2.51)	0.99 (0.59; 1.66)
Affective disorders	Metoprolol (moderate)	1.36 (1.31; 1.41)	1.06 (1.04; 1.08)
	Atenolol (low)	1.19 (1.13; 1.25)	1.05 (1.02; 1.07)
	Bisoprolol (moderate)	1.54 (1.44; 1.66)	1.16 (1.11; 1.21)
	Propranolol (high)	1.87 (1.70; 2.04)	1.21 (1.15; 1.27)
	Sotalol (Low)	1.72 (1.37; 2.16)	1.09 (0.95; 1.24)
	Labetalol (Low)	1.08 (0.66; 1.77)	1.07 (0.86; 1.33)
	Carvedilol (Moderate)	1.37 (1.13; 1.66)	1.08 (0.97; 1.19)
Bipolar disorders	Metoprolol (moderate)	1.41 (1.13; 1.77)	1.02 (0.88; 1.18)
	Atenolol (low)	1.08 (0.77; 1.51)	0.92 (0.78; 1.09)
	Bisoprolol (moderate)	1.43 (0.87; 2.35)	0.83 (0.58; 1.20)
	Propranolol (high)	3.06 (1.97; 4.73)	1.18 (0.86; 1.60)
	Sotalol (Low)	0.97 (1.14; 6.90)	1.38 (0.61; 3.10)
	Labetalol (Low)	2.49 (0.35; 17.7)	1.33 (0.42; 4.17)
	Carvedilol (Moderate)	1.56 (0.50; 4.84)	1.60 (0.92; 2.78)
Depressive disorders	Metoprolol (moderate)	1.36 (1.31; 1.41)	1.07 (1.05; 1.09)
	Atenolol (low)	1.19 (1.13; 1.25)	1.05 (1.02; 1.08)
	Bisoprolol (moderate)	1.55 (1.44; 1.67)	1.17 (1.12; 1.23)
	Propranolol (high)	1.87 (1.71; 2.05)	1.22 (1.16; 1.28)
	Sotalol (Low)	1.71 (1.36; 2.16)	1.11 (0.97; 1.28)
	Labetalol (Low)	1.19 (0.74; 1.92)	1.08 (0.87; 1.34)
	Carvedilol (Moderate)	1.37 (1.12; 1.66)	1.07 (0.96; 1.18)
Anxiety disorders	Metoprolol (moderate)	1.48 (1.44; 1.52)	1.08 (1.06; 1.10)
	Atenolol (low)	1.19 (1.14; 1.24)	1.09 (1.06; 1.11)
	Bisoprolol (moderate)	1.58 (1.49; 1.68)	1.16 (1.12; 1.21)
	Propranolol (high)	2.29 (2.13; 2.46)	1.39 (1.34; 1.44)
	Sotalol (Low)	1.40 (1.11; 1.75)	1.20 (1.07; 1.34)
	Labetalol (Low)	0.89 (0.60; 1.32)	1.26 (1.10; 1.45)
	Carvedilol (Moderate)	1.28 (1.08; 1.52)	0.97 (0.89; 1.06)
Sleep disorders	Metoprolol (moderate)	1.33 (1.29; 1.38)	1.07 (1.05; 1.09)
	Atenolol (low)	0.98 (0.93; 1.04)	1.06 (1.04; 1.08)
	Bisoprolol (moderate)	1.71 (1.61; 1.81)	1.26 (1.22; 1.30)
	Propranolol (high)	1.54 (1.41; 1.68)	1.13 (1.09; 1.18)
	Sotalol (Low)	1.55 (1.26; 1.91)	1.39 (1.27; 1.53)
	Labetalol (Low)	1.27 (0.83, 1.96)	0.95 (0.79; 1.15)
	Carvedilol (Moderate)	1.30 (1.08; 1.55)	1.18 (1.10; 1.27)

Model C: Adjusted for Year of birth, Age at Hypertension, Education, Type of neighborhood, Country of birth, Diabetes mellitus, Thyroid disorders, Opioids, Neuroleptics, Anxiolytics and sleep aids, Antidepressive, Metformin, and Statins. All hypertensive drugs were included in the same model.

Degree of lipophilicity is classified as high, moderate or low (i.e. hydrophilic) [30, 31].

### Co-sibling design

When conducting co-sibling analysis involving siblings discordant for AHT for respective drug classes to adjust for familial factors, the confidence intervals were wider due to a limited number of discordant siblings for many AHT drugs and psychiatric outcomes (Table S12). However, the effect size was still high for several outcomes indicating limited effect of adjustments for familial factors (Table S12). The strongest association with substance abuse disorders in siblings, during the first two years of follow-up, was observed for BBs (HR = 1.55, 95% CI 1.26-1.91) (Table S12).

## Discussion

The present study shows an association between AHT, and several psychiatric disorders. The associations are transiently strongest in the first two years of follow-up, but many associations remain after longer follow-up albeit weaker. However, it is important to emphasize that the findings reflect associations rather than causal relationships, as residual confounding could not be fully accounted for despite the inclusion of a co-sibling design; such as design may provide a stronger support for a causal relationship between certain AHT and APRs. BBs were the “worst” being associated with all included psychiatric disorders except bipolar disorders. BBs seem to have an impact on the brain, especially the high lipophilic beta-blocker propranolol though also more hydrophilic betablockers such as sotalol may be associated with APRs. This has been described in previous scientific literature, and it is also confirmed by our results in the study [13, 28].

Only treatment with BBs was significantly associated with psychotic disorders. Overall, BBs presented the highest risk for APRs. There was no large difference between the other antihypertensive drugs and the association with increased risk for APRs. However, CCBs had a slightly higher risk for psychiatric disorders compared to diuretics, ACEI, and ARB – that all had only minor differences between each other regarding associations with different psychiatric disorders (Table 2). Similar results were presented in an American study that compared CCBs with other AHTs [11]. Their results lead to the conclusion that ARBs were most advantageous regarding the increased risk for psychiatric disorders, while BBs had the least advantage [11], like the present study. Their study included patients aged 18–90 years, differing from our study population’s age inclusion criteria of 40–70 years. Including younger patients may introduce bias, as secondary hypertension is more common, for instance due to genetic mutations [25]. The older population suffers from multimorbidity [26].

Imidazoline receptor agonists and alpha-adrenoreceptor antagonists were prescribed and used by a very small part of the study population compared to other included antihypertensive drugs limiting the conclusion to be drawn. Still, sleep disorders showed a significantly increased risk during 0-2 years of follow-up with imidazoline receptor agonists and alpha-adrenoreceptor antagonists (Model C in Table 2). This is supported by literature indicating that these drugs may impact sleep [32].

Adjustments were made for diabetes mellitus and thyroid disorders because these disorders increase the risk for cardiovascular events and adverse psychiatric reactions [33–35]. Due to the limited numbers of discordant sibling pairs the co-sibling design should be interpretated with caution. Still there were associations between several APRs and different AHT drugs during the first two years of follow-up in the co-sibling design.

### Strengths

Our study’s strength lies in its large registry-based sample. The registries provided detailed data on drug collection, education, and country of birth, reducing bias. We included a generally healthy population with primary care diagnosed hypertension with adjustments made for multiple confounders. Moreover, patients with psychiatric and cardiovascular disorders were excluded before baseline to decrease risk for confounding by indication bias. The study reflects real-world clinical exposure, making findings applicable to practice. A co-sibling analysis may control for shared family background and 50% of genetic effects [18]. High registry coverage in Sweden ensures our findings are generalizable to the Swedish population. Notably, antidepressant, anxiolytic, and sleep aid prescriptions were twice as high in females, which is aligned with national statistics (Swedish National Board of Health and Welfare).

### Limitations

One of the limitations in registry studies is missing information about lifestyle factors and compliance. However, we adjusted for educational level and type of neighborhood, but residual confounding may still exist. We could only determine whether the participant collected the prescribed medication or not. It cannot be excluded that patients may had undiagnosed psychiatric disorders already before hypertension treatment. It is unclear whether substance abuse disorders resulted from high blood pressure, as participants may have had a latent, not diagnosed history of substance abuse disorders, prior to the study. Moreover, as the data were derived from registries with limited access to certain information, alternative study designs would be valuable to confirm the observed increased risk of APRs.

## Conclusion

Our study shows an association with substance abuse disorders, depression disorders, affective disorders, anxiety disorders, and sleep disorders, and treatment with beta-blockers, calcium channel blockers, angiotensin-converting enzyme inhibitors, and angiotensin receptor blockers. Especially lipophilic beta-blockers followed by calcium channel blockers, are associated with adverse psychiatric reactions, while diuretics, angiotensin-converting enzyme inhibitors, and angiotensin receptor blockers have the weakest associations. The association for adverse psychiatric reactions is transiently highest during the first two years after diagnosis of hypertension and decreases thereafter. These findings may be applicable to general clinical practice as they are based on real-world data from a population clinically exposed to antihypertensive drugs. However, inference is limited to associations rather than causation due to residual confounding that cannot be fully accounted for.

## Summary

### What is known about the topic

- Previous studies have shown inconsistent associations between antihypertensive treatment and psychiatric outcomes, with mixed results across drug classes.
- Beta-blockers have been linked to sleep disturbances, while evidence for depression or anxiety remains inconsistent, and some data suggest differences between antihypertensive drug classes.

### What this study adds

- This Swedish primary care–based registry cohort study examines associations between seven antihypertensive drug classes and seven psychiatric disorders in drug-naive patients with hypertension and no prior psychiatric or cardiovascular disease.
- Associations were observed for substance abuse, depressive, affective, anxiety, and sleep disorders, with stronger associations for lipophilic beta-blockers and calcium channel blockers, weaker associations for diuretics, angiotensin-converting enzyme inhibitors, and angiotensin receptor blockers, and the highest risk during the first two years after diagnosis.

## Supplementary information

Supplementary material

## Data Availability

The data supporting the findings of this study are available from the corresponding author upon reasonable request.
