# Supplementary material for: Antihypertensive treatment and adverse psychiatric reactions in primary health care
Source: J Hum Hypertens. 2026 Aug 27;40(11):865–72. doi: 10.1038/s41371-026-01212-y (PMC13747804; doi:10.1038/s41371-026-01212-y)
Supplement: Supplementary file 1 [file 41371_2026_1212_MOESM1_ESM.docx]

**Supplemental online content**

Curic E, Ohlson H, Kendler K, Sundquist J, Sundquist K, Reis M, Zöller B. Antihypertensive Treatment and Adverse Psychiatric Reactions in Primary Health Care.

**Table S1.** Details on ICD-codes for the diseases in the exclusion criteria, diagnoses from the National Patient Register or the nationwide primary care register. Abbreviation: ICD = International Classification of Disease Code

**Table S2.** International Statistical Classification of Diseases 10th Revision (ICD-10) codes for the seven studied psychiatric outcomes.

**Table S3.** ATC-codes for the predictors, antihypertensive drugs. Abbreviation: ATC = Anatomical Therapeutic Chemical Classification System

**Table S4.** International Statistical Classification of Diseases 10th Revision (ICD-10) and ATC codes for the adjusting variables in Model A, Model B and Model C.

**Table S5.** Definition of education and neighborhood variables.

**Table S6.** Median time to treatment initiation across outcome datasets

**Table S7.** Number of concomitant antihypertensive medications among the included patients.

**Table S8.** Cox regression models with time to outcome and AHT as a time-dependent variable in females. Details of the models are described below the table. Abbreviation: AHT = Antihypertensive Treatment

**Table S9.** Cox regression models with time to outcome and AHT as a time-dependent variable in males. Details of the models are described below the table.

**Table S10.** Cox regression models with time to outcome and AHT as a time-dependent variable in both females and males. Details of the models are described below the table.

**Table S11.** Bonferroni adjustment in Model A, Model B, and Model C. Significant outcomes are marked with an asterisk (*). Details of the models are described below the table.

**Table S12.** Co-Sibling analysis.

These supplementary materials has been provided by the authors to offer additional information about their work.

| **Table S1. International Statistical Classification of Diseases 10th Revision (ICD-10) codes for the diseases in the exclusion criteria, diagnoses from the National Patient Register or the nationwide primary care register:** | |
| --- | --- |
| Psychiatric disorders | F00-F48, F51, G30, G31, G47 |
| Coronary heart diseases | I20-I25 |
| Atrial fibrillation | I48 |
| Heart failure | I50 |
| Cerebrovascular diseases | I60-I69 |
| Prostate diseases | C61, N40, N41, N42, D075, D291, D400, N510, Q554, Z125 |

| **Table S2. International Statistical Classification of Diseases 10th Revision (ICD-10) codes for the seven studied psychiatric outcomes.** | |
| --- | --- |
| Outcomes  ICD-codes | |
| Substance abuse disorders | F10-F19 |
| Psychotic disorders | F20-F29 |
| Bipolar disorders | F30, F31 |
| Depressive disorders | F32, F33 |
| Affective disorders | F30-F39 |
| Anxiety disorders | F40-F48 |
| Sleep disorders | F51, G47 |

| **Table S3. Anatomical Therapeutic Chemical (ATC) codes for the predictors, antihypertensive drugs.** | |
| --- | --- |
| Calcium Channel Blocker (CCB) | C08, C07FB, C09BB, C09DB, C09DX01 |
| Diuretics | C03, C02L, C09BA, C09DA, C09DX01 |
| Angiotensin-Converting Enzyme inhibitors (ACE inhibitors) | C09A, C09B |
| Angiotensin II Receptor Blockers (ARB) | C09C, C09D |
| Beta blockers (BBs) | C07 |
| Imidazoline receptor agonists | C02AC |
| Alpha-adrenoreceptor antagonists | C02CA |

| **Table S4. International Statistical Classification of Diseases 10th Revision (ICD-10) and Anatomical Therapeutic Chemical (ATC) codes for the adjusting variables in Model A, Model B and Model C.** | |
| --- | --- |
| Adjusting variables | Definition, ICD-codes, or ATC-codes |
| Age | Years |
| Sex | Female or male |
| Education | Low, mid, high according to Supplementary Table 5 |
| Neighborhood deprivation | Low, mid, high according to Supplementary Table 5 |
| Country of birth | Born in Sweden with 0, 1, or 2 parents born outside Sweden or born outside Sweden |
| Diabetes mellitus | E10-E14 |
| Thyroid disorders | E00-E07 |
| Opioids | N02A |
| Neuroleptics | N05A |
| Anxiolytica and sleep aids | N05B, N05C |
| Antidepressants | N06A |
| Metformin | [A10BA02](https://www.fass.se/LIF/atcregister?atcCode=A10BA02) |
| Statins | C10AA, C10B (not C10BA10) |

| **Table S5. Definition of education and type of neighborhood variables.** | |
| --- | --- |
| Type of Neighborhood | For every year an individual is registered at a specific DESO area (Swedish abbreviation for demographic statistical area). The DeSO areas divides Sweden into 5,983 areas and have between 700 and 2,700 inhabitants. The division takes into account the geographical conditions so that the boundaries follow, for example, streets, waterways and railways. Important building blocks used to create DeSO are urban areas and electoral districts. For each of the DeSO area we created a neighborhood social deprivation index based on register data for all residents in the neighborhood aged 25-64. We used deprivation indicators used by past studies to characterize neighborhood environments and then used a principal component analysis to calculate a z-score. The following four variables were included: low educational status (defined as less than 10 years of formal education); low income (from all sources, including that from interest and dividends, which was defined as less than 50% of individual median income); unemployment (defined as not employed; excluding full-time students, those completing compulsory military service, and early retirees); and social welfare assistance. We divided the deprivation score into three groups: low (below 1 Standard deviation [sd] of the mean), mid (-1 sd to 1 sd of the mean) and high deprivation as above one sd from the mean value. |
| Education | In the registers the education variable is coded as follows:  1 Pre-high school < 9 years  2 High School 9 years  3 Upper Secondary School < 3 years  4 Upper Secondary School 3 years  5 Post-secondary education < 3years  6 Post-secondary education 3 years or more  7 Research education (PhD).  From this we created a three-level variable: Low (1.2), mid (3-4) and high (5-7). |

| **Table S6. Median time (years) to treatment initiation across outcome datasets** | | |
| --- | --- | --- |
|  | Min | Max |
| Calcium channel blocker | 2.03 (2.01; 2.05) | 2.29 (2.27; 3.30) |
| Diuretics | 0.52 (0.50, 0.53) | 0.68 (0.67; 0.70) |
| Angiotensin-Converting Enzyme inhibitors (ACE inhibitors) | 0.23 (0.22, 0.25) | 0.37 (0.36; 0.38) |
| Angiotensin II Receptor Blockers (ARB) | 1.08 (1.07; 1.10) | 1.31 (1.30; 1.33) |
| Beta blockers (BB) | 0.25 (0.24; 0.27) | 0.45 (0.44; 0.47) |
| Imidazoline receptor agonists | 2.97 (2.73; 3.21) | 3.57 (3.39; 3.80) |
| Alpha-adrenoreceptor antagonists | 4.06 (3.98; 4.15) | 4.49 (4.41, 4.58) |

| **Table S7. Number of concomitant antihypertensive medications among the included patients** | |
| --- | --- |
| *0* | *9.1%* |
| *1* | *18.1%* |
| *2* | *23.5%* |
| *3* | *22.6%* |
| *4* | *17.8%* |
| *5* | *8.1%* |
| *6* | *0.8%* |
| *7* | *0.1%* |

| **Table S8. Bonferroni adjustment in Model A, Model B, and Model C. Bonferroni significant outcomes marked with an asterisk (*).** | | | | | | | |
| --- | --- | --- | --- | --- | --- | --- | --- |
| MALES AND FEMALES |  | MODEL A | | MODEL B | | MODEL C | |
|  |  | 0-2 years | 2 + years | 0-2 years | 2 + years | 0-2 years | 2 + years |
| OUTCOME | PREDICTOR | HR 95 % CI | HR 95 % CI | HR 95 % CI | HR 95 % CI | HR 95 % CI | HR 95 % CI |
| Substance abuse disorders | Calcium Channel Blocker (CCB) | <.0001* | <.0001* | <.0001* | <.0001* | <.0001* | <.0001* |
|  | Diuretics | <.0001* | <.0001* | <.0001* | <.0001* | <.0001* | <.0001* |
|  | Angiotensin-Converting Enzyme inhibitors (ACE inhibitors) | <.0001* | <.0001* | <.0001* | <.0001* | <.0001* | <.0001* |
|  | Angiotensin II Receptor Blockers (ARB) | <.0001* | 0.0021 | <.0001* | <.0001* | 0.0320 | <.0001* |
|  | Beta blockers (BBs) | <.0001* | <.0001* | <.0001* | <.0001* | <.0001* | <.0001* |
|  | Imidazoline receptor agonists | <.0001* | 0.477 | 0.0004* | 0.8799 | 0.0106 | 0.8639 |
|  | Alpha-adrenoreceptor antagonists | 0.3014 | 0.0020 | 0.1692 | <.0001* | <.0001* | <.0001* |
|  |  |  |  |  |  |  |  |
| Psychotic disorders | Calcium Channel Blocker (CCB) | 0.4704 | 0.9673 | 0.5066 | 0.6608 | 0.5518 | 0.9703 |
|  | Diuretics | 0.7850 | 0.2900 | 0.8117 | 0.0499 | 0.9591 | 0.1974 |
|  | Angiotensin-Converting Enzyme inhibitors (ACE inhibitors) | 0.8193 | 0.6126 | 0.7356 | 0.1767 | 0.2719 | 0.1316 |
|  | Angiotensin II Receptor Blockers (ARB) | 0.0055 | 0.0015 | 0.0057 | 0.0001* | 0.0016 | 0.0003* |
|  | Beta blockers (BBs) | <.0001* | <.0001* | <.0001* | 0.0046 | <.0001* | 0.0022 |
|  | Imidazoline receptor agonists | 0.7104 | 0.6845 | 0.8364 | 0.9986 | 0.9286 | 0.7891 |
|  | Alpha-adrenoreceptor antagonists | 0.0592 | 0.2499 | 0.1056 | 0.1547 | 0.1198 | 0.1895 |
|  |  |  |  |  |  |  |  |
| Affective disorders | Calcium Channel Blocker (CCB) | <.0001* | <.0001* | <.0001* | <.0001* | <.0001* | <.0001* |
|  | Diuretics | <.0001* | <.0001* | <.0001* | <.0001* | <.0001* | <.0001* |
|  | Angiotensin-Converting Enzyme inhibitors (ACE inhibitors) | <.0001* | 0.0148 | <.0001* | 0.4992 | 0.0024 | 0.2271 |
|  | Angiotensin II Receptor Blockers (ARB) | <.0001* | <.0001* | <.0001* | <.0001* | <.0001* | 0.0002* |
|  | Beta blockers (BBs) | <.0001* | <.0001* | <.0001* | <.0001* | <.0001* | <.0001* |
|  | Imidazoline receptor agonists | 0.0006 | <.0001* | 0.0116 | 0.0429 | 0.2686 | 0.1946 |
|  | Alpha-adrenoreceptor antagonists | <.0001* | <.0001* | <.0001* | 0.0081 | 0.0129 | 0.1107 |
|  |  |  |  |  |  |  |  |
| Bipolar disorders | Calcium Channel Blocker (CCB) | 0.4646 | 0.4252 | 0.3898 | 0.1291 | 0.2723 | 0.2924 |
|  | Diuretics | 0.0273 | 0.2848 | 0.0388 | 0.8242 | 0.0756 | 0.6025 |
|  | Angiotensin-Converting Enzyme inhibitors (ACE inhibitors) | 0.8188 | 0.8188 | 0.9092 | 0.0026 | 0.5418 | 0.0039 |
|  | Angiotensin II Receptor Blockers (ARB) | 0.8476 | 0.9550 | 0.8533 | 0.3740 | 0.4492 | 0.2890 |
|  | Beta blockers (BBs) | <.0001* | 0.0156 | 0.0004* | 0.5339 | 0.0018 | 0.4518 |
|  | Imidazoline receptor agonists | 0.8897 | 0.5671 | 0.9505 | 0.9957 | 0.9800 | 0.9493 |
|  | Alpha-adrenoreceptor antagonists | 0.6299 | 0.7150 | 0.5113 | 0.9770 | 0.4873 | 0.8613 |
|  |  |  |  |  |  |  |  |
| Depressive disorders | Calcium Channel Blocker (CCB) | <.0001* | <.0001* | <.0001* | <.0001* | <.0001* | 0.0001* |
|  | Diuretics | <.0001* | <.0001* | <.0001* | <.0001* | <.0001* | <.0001* |
|  | Angiotensin-Converting Enzyme inhibitors (ACE inhibitors) | <.0001* | 0.0060 | <.0001* | 0.8219 | 0.0007 | 0.4027 |
|  | Angiotensin II Receptor Blockers (ARB) | <.0001* | <.0001* | <.0001* | <.0001* | <.0001* | 0.0004* |
|  | Beta blockers (BBs) | <.0001* | <.0001* | <.0001* | <.0001* | <.0001* | <.0001* |
|  | Imidazoline receptor agonists | 0.0003* | <.0001* | 0.0056 | 0.0400 | 0.1967 | 0.1913 |
|  | Alpha-adrenoreceptor antagonists | <.0001* | <.0001* | <.0001* | 0.0087 | 0.0056 | 0.1289 |
|  |  |  |  |  |  |  |  |
| Anxiety disorders | Calcium Channel Blocker (CCB) | <.0001* | <.0001* | <.0001* | 0.0013 | <.0001* | 0.0585 |
|  | Diuretics | <.0001* | <.0001* | <.0001* | 0.5648 | 0.8409 | 0.0864 |
|  | Angiotensin-Converting Enzyme inhibitors (ACE inhibitors) | <.0001* | <.0001* | <.0001* | <.0001* | <.0001* | <.0001* |
|  | Angiotensin II Receptor Blockers (ARB) | <.0001* | <.0001* | <.0001* | <.0001* | <.0001* | <.0001* |
|  | Beta blockers (BBs) | <.0001* | <.0001* | <.0001* | <.0001* | <.0001* | <.0001* |
|  | Imidazoline receptor agonists | <.0001* | <.0001* | 0.0009 | 0.0004* | 0.0752 | 0.0009 |
|  | Alpha-adrenoreceptor antagonists | <.0001* | 0.5697 | <.0001* | 0.6284 | 0.1165 | 0.1606 |
|  |  |  |  |  |  |  |  |
| Sleep disorders | Calcium Channel Blocker (CCB) | <.0001* | <.0001* | <.0001* | <.0001* | <.0001* | <.0001* |
|  | Diuretics | <.0001* | <.0001* | <.0001* | <.0001* | <.0001* | <.0001* |
|  | Angiotensin-Converting Enzyme inhibitors (ACE inhibitors) | <.0001* | 0.8957 | <.0001* | 0.1182 | <.0001* | 0.0209 |
|  | Angiotensin II Receptor Blockers (ARB) | <.0001* | <.0001* | <.0001* | <.0001* | <.0001* | <.0001* |
|  | Beta blockers (BBs) | <.0001* | <.0001* | <.0001* | <.0001* | <.0001* | <.0001* |
|  | Imidazoline receptor agonists | <.0001* | <.0001* | <.0001* | 0.0388 | <.0001* | 0.4631 |
|  | Alpha-adrenoreceptor antagonists | <.0001* | <.0001* | <.0001* | 0.0170 | <.0001* | 0.9960 |
| Model A: Adjusting for Year of birth, Age at Hypertension, Education, Type of neighborhood, Country of birth. Model B: Adjusting for Year of birth, Age at Hypertension, Education, Type of neighborhood, Country of birth, Diabetes mellitus, Thyroid disorders, Opioids, Neuroleptics, Anxiolytics and sleep aids, Antidepressive, Metformin, Statins. Model C: Adjusted for Year of birth, Age at Hypertension, Education, Type of neighborhood, Country of birth, Diabetes mellitus, Thyroid disorders, Opioids, Neuroleptics, Anxiolytics and sleep aids, Antidepressive, Metformin, and Statins. All hypertensive drugs were included in the same model. | | | | | | | |

| **Table S9. Cox regression models with time to outcome and AHT as a time-dependent variable in females** | | | | | | | |
| --- | --- | --- | --- | --- | --- | --- | --- |
|  |  | MODEL A | | MODEL B | | MODEL C | |
|  |  | 0-2 years | 2 + years | 0-2 years | 2 + years | 0-2 years | 2 + years |
| OUTCOME | PREDICTOR | HR 95 % CI | HR 95 % CI | HR 95 % CI | HR 95 % CI | HR 95 % CI | HR 95 % CI |
| Substance abuse disorders | Calcium Channel Blocker (CCB) | 1.45 (1.38; 1.52) | 1.25 (1.21; 1.30) | 1.44 (1.37; 1.51) | 1.22 (1.17; 1.27) | 1.35 (1.28; 1.41) | 1.19 (1.14; 1.24) |
|  | Diuretics | 1.29 (1.22; 1.36) | 1.21 (1.17; 1.26) | 1.28 (1.22; 1.35) | 1.15 (1.11; 1.20) | 1.16 (1.10; 1.22) | 1.12 (1.08; 1.16) |
|  | Angiotensin-Converting Enzyme inhibitors (ACE inhibitors) | 1.34 (1.27; 1.41) | 1.14 (1.10; 1.18) | 1.36 (1.29; 1.43) | 1.12 (1.08; 1.16) | 1.27 (1.21; 1.34) | 1.08 (1.05; 1.12) |
|  | Angiotensin II Receptor Blockers (ARB) | 1.14 (1.08; 1.20) | 1.00 (0.96; 1.04) | 1.14 (1.08; 1.20) | 0.96 (0.93; 1.00) | 1.06 (1.00; 1.12) | 0.94 (0.90; 0.97) |
|  | Beta blockers (BBs) | 1.38 (1.31; 1.46) | 1.27 (1.22; 1.31) | 1.37 (1.30; 1.44) | 1.21 (1.17; 1.25) | 1.28 (1.21; 1.35) | 1.18 (1.14; 1.22) |
|  | Imidazoline receptor agonists | 1.83 (1.14; 2.95) | 1.07 (0.75; 1.55) | 1.73 (1.08; 2.79) | 0.96 (0.67; 1.38) | 1.49 (0.93; 2.40) | 0.93 (0.64; 1.34) |
|  | Alpha-adrenoreceptor antagonists | 1.01 (0.75; 1.36) | 0.95 (0.76; 1.19) | 0.91 (0.73; 1.32) | 0.91 (0.73; 1.14) | 0.77 (0.57; 1.05) | 0.82 (0.65; 1.03) |
|  |  |  |  |  |  |  |  |
| Psychotic disorders | Calcium Channel Blocker (CCB) | 1.01 (0.83; 1.23) | 1.10 (0.96; 1.27) | 1.00 (0.83; 1.22) | 1.06 (0.92; 1.23) | 0.99 (0.81; 1.21) | 1.07 (0.93; 1.24) |
|  | Diuretics | 0.98 (0.82; 1.18) | 0.95 (0.84; 1.08) | 0.98 (0.81; 1.18) | 0.91 (0.80; 1.03) | 0.94 (0.77; 1.14) | 0.90 (0.79; 1.03) |
|  | Angiotensin-Converting Enzyme inhibitors (ACE inhibitors) | 1.00 (0.83; 1.21) | 1.01 (0.89; 1.14) | 0.99 (0.82; 1.20) | 0.95 (0.84; 1.08) | 0.97 (0.80; 1.18) | 0.96 (0.84; 1.09) |
|  | Angiotensin II Receptor Blockers (ARB) | 0.85 (0.68; 1.05) | 0.99 (0.86; 1.13) | 0.84 (0.67; 1.04) | 0.94 (0.83; 1.07) | 0.82 (0.66; 1.03) | 0.95 (0.83; 1.09) |
|  | Beta blockers (BBs) | 1.48 (1.24; 1.77) | 1.20 (1.06; 1.36) | 1.46 (1.22; 1.75) | 1.14 (1.00; 1.29) | 1.49 (1.24; 1.79) | 1.14 (1.00; 1.29) |
|  | Imidazoline receptor agonists | 2.40 (0.60; 9.64) | 1.20 (0.39; 3.72) | 2.13 (0.53; 8.58) | 1.01 (0.32; 3.16) | 1.89 (0.43; 8.18) | 0.99 (0.32; 3.16) |
|  | Alpha-adrenoreceptor antagonists | 1.59 (0.71; 3.56) | 1.20 (0.62; 2.32) | 1.46 (0.66; 3.25) | 1.09 (0.56; 2.11) | 1.33 (0.58; 3.08) | 1.06 (0.54; 2.08) |
|  |  |  |  |  |  |  |  |
| Affective disorders | Calcium Channel Blocker (CCB) | 1.21 (1.17; 1.25) | 1.10 (1.07; 1.13) | 1.20 (1.16; 1.24) | 1.05 (1.02; 1.08) | 1.15 (1.11; 1.19) | 1.03 (1.00; 1.06) |
|  | Diuretics | 1.17 (1.13; 1.21) | 1.18 (1.15; 1.21) | 1.16 (1.12; 1.20) | 1.08 (1.06; 1.11) | 1.07 (1.03; 1.11) | 1.07 (1.04; 1.09) |
|  | Angiotensin-Converting Enzyme inhibitors (ACE inhibitors) | 1.07 (1.04; 1.11) | 1.03 (1.00; 1.05) | 1.09 (1.06; 1.13) | 1.00 (0.97; 1.02) | 1.05 (1.02; 1.09) | 0.99 (0.97; 1.02) |
|  | Angiotensin II Receptor Blockers (ARB) | 1.15 (1.11; 1.19) | 1.12 (1.10; 1.15) | 1.15 (1.11; 1.19) | 1.05 (1.03; 1.08) | 1.10 (1.06; 1.14) | 1.04 (1.01; 1.07) |
|  | Beta blockers (BBs) | 1.32 (1.28; 1.37) | 1.16 (1.13; 1.19) | 1.29 (1.25; 1.34) | 1.06 (1.03; 1.08) | 1.25 (1.21; 1.30) | 1.04 (1.02; 1.07) |
|  | Imidazoline receptor agonists | 1.74 (1.27; 2.39) | 1.50 (1.22; 1.85) | 1.57 (1.14; 2.16) | 1.23 (1.00; 1.52) | 1.35 (0.98; 1.86) | 1.18 (0.95; 1.45) |
|  | Alpha-adrenoreceptor antagonists | 1.40 (1.19; 1.66) | 1.20 (1.05; 1.38) | 1.32 (1.12; 1.57) | 1.13 (0.98; 1.29) | 1.16 (0.98; 1.38) | 1.07 (0.94; 1.23) |
|  |  |  |  |  |  |  |  |
| Bipolar disorders | Calcium Channel Blocker (CCB) | 1.05 (0.80; 1.36) | 1.09 (0.89; 1.33) | 1.02 (0.78; 1.33) | 1.01 (0.83; 1.24) | 1.01 (0.77; 1.31) | 1.04 (0.85; 1.27) |
|  | Diuretics | 1.15 (0.91; 1.46) | 1.04 (0.87; 1.23) | 1.14 (0.90; 1.44) | 0.94 (0.79; 1.12) | 1.10 (0.86; 1.41) | 0.95 (0.80; 1.13) |
|  | Angiotensin-Converting Enzyme inhibitors (ACE inhibitors) | 0.91 (0.70; 1.18) | 0.93 (0.78; 1.12) | 0.91 (0.70; 1.18) | 0.89 (0.74; 1.06) | 0.88 (0.67; 1.14) | 0.90 (0.75; 1.08) |
|  | Angiotensin II Receptor Blockers (ARB) | 0.96 (0.73; 1.26) | 1.03 (0.85; 1.24) | 0.95 (0.72; 1.25) | 0.95 (0.78; 1.14) | 0.91 (0.69; 1.21) | 0.95 (0.79; 1.15) |
|  | Beta blockers (BBs) | 1.54 (1.22; 1.94) | 1.29 (1.09; 1.53) | 1.50 (1.19; 1.88) | 1.16 (0.98; 1.37) | 1.48 (1.17; 1.88) | 1.17 (0.99; 1.38) |
|  | Imidazoline receptor agonists | - | 2.74 (0.88; 8.53) | - | 2.03 (0.65; 6.35) | - | 2.31 (0.76; 7.05) |
|  | Alpha-adrenoreceptor antagonists | - | 0.63 (0.16; 2.54) | - | 0.55 (0.14; 2.21) | - | 0.50 (0.13; 1.95) |
|  |  |  |  |  |  |  |  |
| Depressive disorders | Calcium Channel Blocker (CCB) | 1.22 (1.18; 1.27) | 1.10 (1.07; 1.13) | 1.21 (1.17; 1.26) | 1.05 (1.02; 1.08) | 1.16 (1.12; 1.20) | 1.03 (1.00; 1.06) |
|  | Diuretics | 1.18 (1.14; 1.22) | 1.18 (1.16; 1.21) | 1.16 (1.12; 1.20) | 1.09 (1.06; 1.11) | 1.08 (1.04; 1.12) | 1.07 (1.05; 1.10) |
|  | Angiotensin-Converting Enzyme inhibitors (ACE inhibitors) | 1.08 (1.04; 1.12) | 1.03 (1.00; 1.05) | 1.10 (1.06; 1.14) | 1.00 (0.98; 1.03) | 1.06 (1.02; 1.10) | 0.99 (0.97; 1.02) |
|  | Angiotensin II Receptor Blockers (ARB) | 1.15 (1.11: 1.19) | 1.12 (1.09; 1.15) | 1.15 (1.11: 1.19) | 1.05 (1.03; 1.08) | 1.10 (1.06: 1.14) | 1.04 (1.01; 1.06) |
|  | Beta blockers (BBs) | 1.31 (1.27; 1.36) | 1.17 (1.14; 1.20) | 1.29 (1.24; 1.33) | 1.07 (1.04; 1.09) | 1.24 (1.20; 1.29) | 1.05 (1.03; 1.08) |
|  | Imidazoline receptor agonists | 1.79 (1.30; 2.46) | 1.48 (1.19; 1.83) | 1.62 (1.18; 2.23) | 1.22 (0.99; 1.51) | 1.39 (1.00; 1.91) | 1.16 (0.94; 1.44) |
|  | Alpha-adrenoreceptor antagonists | 1.41 (1.19; 1.67) | 1.20 (1.05; 1.38) | 1.34 (1.13; 1.58) | 1.13 (0.98; 1.30) | 1.17 (0.97; 1.39) | 1.07 (0.93; 1.23) |
|  |  |  |  |  |  |  |  |
| Anxiety disorders | Calcium Channel Blocker (CCB) | 1.29 (1.26; 1.32) | 1.06 (1.04; 1.09) | 1.28 (1.25; 1.31) | 1.03 (1.01; 1.05) | 1.22 (1.18; 1.25) | 1.02 (1.00; 1.04) |
|  | Diuretics | 1.13 (1.10; 1.16) | 1.07 (1.05; 1.09) | 1.11 (1.08; 1.14) | 1.01 (0.99; 1.03) | 1.00 (0.97; 1.04) | 0.99 (0.97; 1.02) |
|  | Angiotensin-Converting Enzyme inhibitors (ACE inhibitors) | 1.09 (1.07; 1.12) | 0.96 (0.95; 0.98) | 1.11 (1.08; 1.14) | 0.96 (0.94; 0.98) | 1.07 (1.04; 1.10) | 0.97 (0.95; 0.99) |
|  | Angiotensin II Receptor Blockers (ARB) | 1.19 (1.15; 1.21) | 1.10 (1.08; 1.13) | 1.19 (1.16; 1.22) | 1.07 (1.04; 1.09) | 1.13 (1.10; 1.17) | 1.07 (1.05; 1.09) |
|  | Beta blockers (BBs) | 1.52 (1.48; 1.56) | 1.18 (1.16; 1.21) | 1.49 (1.45; 1.53) | 1.11 (1.09; 1.13) | 1.44 (1.41; 1.48) | 1.11 (1.09; 1.13) |
|  | Imidazoline receptor agonists | 1.60 (1.22; 2.10) | 1.52 (1.29; 1.80) | 1.47 (1.12; 1.93) | 1.31 (1.10; 1.56) | 1.26 (0.95; 1.66) | 1.29 (1.08; 1.53) |
|  | Alpha-adrenoreceptor antagonists | 1.29 (1.12; 1.48) | 1.06 (0.95; 1.19) | 1.26 (1.10; 1.45) | 1.02 (0.91; 1.15) | 1.08 (0.93; 1.24) | 0.97 (0.87; 1.09) |
|  |  |  |  |  |  |  |  |
| Sleep disorders | Calcium Channel Blocker (CCB) | 1.24 (1.21; 1.28) | 1.09 (1.07; 1.11) | 1.23 (1.20; 1.27) | 1.05 (1.03; 1.07) | 1.17 (1.14; 1.21) | 1.01 (0.99; 1.04) |
|  | Diuretics | 1.18 (1.14; 1.21) | 1.22 (1.20; 1.25) | 1.16 (1.13; 1.20) | 1.13 (1.11; 1.16) | 1.07 (1.04; 1.11) | 1.10 (1.08; 1.13) |
|  | Angiotensin-Converting Enzyme inhibitors (ACE inhibitors) | 1.07 (1.04; 1.11) | 0.99 (0.97; 1.01) | 1.10 (1.07; 1.14) | 0.98 (0.96; 1.00) | 1.07 (1.04; 1.11) | 0.97 (0.95; 0.99) |
|  | Angiotensin II Receptor Blockers (ARB) | 1.25 (1.21; 1.29) | 1.21; 1.18; 1.23) | 1.25 (1.22; 1.29) | 1.15; 1.13; 1.17) | 1.20 (1.17; 1.24) | 1.13; 1.10; 1.15) |
|  | Beta blockers (BBs) | 1.26 (1.22; 1.31) | 1.21 (1.19; 1.23) | 1.23 (1.19; 1.27) | 1.11 (1.09; 1.13) | 1.18 (1.15; 1.22) | 1.10 (1.07; 1.12) |
|  | Imidazoline receptor agonists | 2.10 (1.63; 2.70) | 1.43 (1.20; 1.71) | 1.92 (1.50; 2.48) | 1.20 (1.01; 1.44) | 1.67 (1.29; 2.15) | 1.14 (0.95; 1.37) |
|  | Alpha-adrenoreceptor antagonists | 1.31 (1.13; 1.51) | 1.07 (0.95; 1.20) | 1.25 (1.08; 1.44) | 1.01 (0.90; 1.13) | 1.07 (0.92; 1.23) | 0.93 (0.83; 1.04) |
| Model A: Adjusting for Year of birth, Age at Hypertension, Education, Type of neighborhood, Country of birth. Model B: Adjusting for Year of birth, Age at Hypertension, Education, Type of neighborhood, Country of birth, Diabetes mellitus, Thyroid disorders, Opioids, Neuroleptics, Anxiolytics and sleep aids, Antidepressive, Metformin, Statins. Model C: Adjusted for Year of birth, Age at Hypertension, Education, Type of neighborhood, Country of birth, Diabetes mellitus, Thyroid disorders, Opioids, Neuroleptics, Anxiolytics and sleep aids, Antidepressive, Metformin, and Statins. All hypertensive drugs were included in the same model. | | | | | | | |

| **Table S10. Cox regression models with time to outcome and AHT as a time-dependent variable in males** | | | | | | | |
| --- | --- | --- | --- | --- | --- | --- | --- |
|  |  | MODEL A | | MODEL B | | MODEL C | |
|  |  | 0-2 years | 2 + years | 0-2 years | 2 + years | 0-2 years | 2 + years |
| OUTCOME | PREDICTOR | HR 95 % CI | HR 95 % CI | HR 95 % CI | HR 95 % CI | HR 95 % CI | HR 95 % CI |
| Substance abuse disorders | Calcium Channel Blocker (CCB) | 1.45 (1.39; 1.51) | 1.18 (1.14; 1.22) | 1.45 (1.39; 1.50) | 1.15 (1.12; 1.19) | 1.33 (1.28; 1.39) | 1.13 (1.09; 1.17) |
|  | Diuretics | 1.30 (1.24; 1.35) | 1.14 (1.10; 1.18) | 1.29 (1.24; 1.35) | 1.11 (1.08; 1.15) | 1.12 (1.07; 1.18) | 1.09 (1.05; 1.13) |
|  | Angiotensin-Converting Enzyme inhibitors (ACE inhibitors) | 1.29 (1.23; 1.34) | 1.08 (1.05; 1.12) | 1.29 (1.24; 1.35) | 1.06 (1.03; 1.10) | 1.19 (1.14; 1.25) | 1.02 (0.99; 1.06) |
|  | Angiotensin II Receptor Blockers (ARB) | 1.09 (1.04; 1.15) | 0.94 (0.91; 0.97) | 1.10 (1.05; 1.15) | 0.91 (0.88; 0.94) | 1.03 (0.98; 1.08) | 0.90 (0.87; 0.93) |
|  | Beta blockers (BBs) | 1.79 (1.71; 1.87) | 1.33 (1.29; 1.37) | 1.76 (1.69; 1.84) | 1.28 (1.24; 1.33) | 1.64 (1.57; 1.72) | 1.27 (1.23; 1.31) |
|  | Imidazoline receptor agonists | 1.80 (1.23; 2.62) | 1.10 (0.81; 1.49) | 1.69 (1.16; 2.46) | 1.00 (0.73; 1.35) | 1.45 (0.99; 2.12) | 1.01 (0.74; 1.38) |
|  | Alpha-adrenoreceptor antagonists | 0.89 (0.74; 1.07) | 0.79 (0.69; 0.96) | 0.86 (0.72; 1.04) | 0.75 (0.66; 0.87) | 0.69 (0.57; 0.84) | 0.71 (0.62; 0.82) |
|  |  |  |  |  |  |  |  |
| Psychotic disorders | Calcium Channel Blocker (CCB) | 1.08 (0.88; 1.33) | 0.87 (0.73; 1.04) | 1.09 (0.89; 1.34) | 0.86 (0.72; 1.03) | 1.09 (0.88; 1.34) | 0.90 (0.75; 1.07) |
|  | Diuretics | 1.07 (0.87; 1.33) | 0.93 (0.79; 1.09) | 1.07 (0.86; 1.32) | 0.90 (0.77; 1.06) | 1.10 (0.88; 1.37) | 1.01 (0.85; 1.19) |
|  | Angiotensin-Converting Enzyme inhibitors (ACE inhibitors) | 0.95 (0.77; 1.18) | 0.94 (0.80; 1.09) | 0.95 (0.76; 1.18) | 0.92 (0.79; 1.07) | 0.83 (0.67; 1.04) | 0.86 (0.73; 1.01) |
|  | Angiotensin II Receptor Blockers (ARB) | 0.71 (0.55; 0.92) | 0.65 (0.54; 0.78) | 0.72 (0.56; 0.94) | 0.64 (0.54; 0.76) | 0.67 (0.52; 0.87) | 0.62 (0.52; 0.75) |
|  | Beta blockers (BBs) | 1.48 (1.20; 1.83) | 1.25 (1.07; 1.46) | 1.46 (1.18; 1.80) | 1.19 (1.02; 1.39) | 1.45 (1.16; 1.80) | 1.22 (1.04; 1.44) |
|  | Imidazoline receptor agonists | - | 1.19 (0.30; 4.78) | - | 0.97 (0.24; 3.97) | - | 1.24 (0.30; 5.13) |
|  | Alpha-adrenoreceptor antagonists | 1.65 (0.86; 3.19) | 0.44 (0.18; 1.08) | 1.57 (0.81; 3.04) | 0.42 (0.18; 1.03) | 1.67 (0.86; 3.25) | 0.46 (0.19; 1.13) |
|  |  |  |  |  |  |  |  |
| Affective disorders | Calcium Channel Blocker (CCB) | 1.28 (1.23; 1.33) | 1.15 (1.11; 1.19) | 1.28 (1.23; 1.33) | 1.10 (1.07; 1.14) | 1.19 (1.14; 1.24) | 1.06 (1.03; 1.10) |
|  | Diuretics | 1.26 (1.21; 1.32) | 1.17 (1.13; 1.20) | 1.25 (1.20; 1.31) | 1.10 (1.07; 1.14) | 1.11 (1.06; 1.17) | 1.06 (1.02; 1.09) |
|  | Angiotensin-Converting Enzyme inhibitors (ACE inhibitors) | 1.06 (1.02; 1.11) | 1.02 (0.99; 1.05) | 1.08 (1.04; 1.13) | 0.99 (0.96; 1.02) | 1.03 (0.98; 1.08) | 0.98 (0.95; 1.02) |
|  | Angiotensin II Receptor Blockers (ARB) | 1.18 (1.13; 1.23) | 1.13 (1.09; 1.17) | 1.18 (1.13; 1.23) | 1.07 (1.03; 1.10) | 1.11 (1.06; 1.16) | 1.04 (1.01; 1.08) |
|  | Beta blockers (BBs) | 1.68 (1.61; 1.75) | 1.33 (1.29; 1.37) | 1.63 (1.56; 1.70) | 1.21 (1.17; 1.25) | 1.55 (1.48; 1.62) | 1.19 (1.15; 1.23) |
|  | Imidazoline receptor agonists | 1.29 (0.82; 2.00) | 1.38 (1.04; 1.81) | 1.13 (0.73; 1.75) | 1.10 (0.84; 1.46) | 0.89 (0.57; 1.39) | 1.01 (0.76; 1.34) |
|  | Alpha-adrenoreceptor antagonists | 1.38 (1.19; 1.61) | 1.21 (1.08; 1.36) | 1.32 (1.14; 1.54) | 1.11 (0.93; 1.25) | 1.12 (0.96; 1.31) | 1.06 (0.94; 1.19) |
|  |  |  |  |  |  |  |  |
| Bipolar disorders | Calcium Channel Blocker (CCB) | 0.82 (0.61; 1.08) | 0.79 (0.64; 0.98) | 0.82 (0.61; 1.09) | 0.77 (0.62; 0.95) | 0.78 (0.59; 1.04) | 0.79 (0.64; 0.99) |
|  | Diuretics | 1.29 (1.00; 1.67) | 1.12 (0.93; 1.36) | 1.27 (0.98; 1.65) | 1.05 (0.87; 1.27) | 1.29 (0.99; 1.68) | 1.20 (0.98; 1.47) |
|  | Angiotensin-Converting Enzyme inhibitors (ACE inhibitors) | 1.07 (0.82; 1.40) | 0.77 (0.63; 0.93) | 1.09 (0.84; 1.42) | 0.75 (0.62; 0.90) | 1.03 (0.79; 1.34) | 0.73 (0.60; 0.89) |
|  | Angiotensin II Receptor Blockers (ARB) | 1.01 (0.75; 1.35) | 0.97 (0.79; 1.19) | 1.01 (0.76; 1.35) | 0.92 (0.76; 1.13) | 0.94 (0.70; 1.26) | 0.86 (0.70; 1.07) |
|  | Beta blockers (BBs) | 1.30 (0.98; 1.71) | 1.04 (0.85; 1.27) | 1.25 (0.94; 1.64) | 0.92 (0.76; 1.12) | 1.19 (0.89; 1.59) | 0.93 (0.76; 1.14) |
|  | Imidazoline receptor agonists | - | - | - | - | - | - |
|  | Alpha-adrenoreceptor antagonists | 1.20 (0.45; 3.19) | 1.33 (0.69; 2.59) | 1.14 (0.43; 3.05) | 1.24 (0.64; 2.42) | 1.17 (0.45; 3.03) | 1.41 (0.72; 2.76) |
|  |  |  |  |  |  |  |  |
| Depressive disorders | Calcium Channel Blocker (CCB) | 1.29 (1.23; 1.34) | 1.15 (1.11; 1.19) | 1.29 (1.24; 1.35) | 1.15 (1.11; 1.19) | 1.20 (1.14; 1.25) | 1.07 (1.03; 1.11) |
|  | Diuretics | 1.27 (1.22; 1.33) | 1.17 (1.13; 1.21) | 1.26 (1.21; 1.32) | 1.11 (1.07; 1.14) | 1.11 (1.06; 1.17) | 1.06 (1.02; 1.09) |
|  | Angiotensin-Converting Enzyme inhibitors (ACE inhibitors) | 1.07 (1.03; 1.12) | 1.03 (0.99; 1.06) | 1.09 (1.04; 1.14) | 1.00 (0.96; 1.03) | 1.04 (0.99; 1.08) | 0.99 (0.96; 1.02) |
|  | Angiotensin II Receptor Blockers (ARB) | 1.19 (1.13; 1.24) | 1.13 (1.09; 1.17) | 1.19 (1.14; 1.25) | 1.07 (1.03; 1.11) | 1.11 (1.06; 1.17) | 1.05 (1.01; 1.08) |
|  | Beta blockers (BBs) | 1.72 (1.64; 1.79) | 1.34 (1.30; 1.39) | 1.67 (1.59; 1.74) | 1.22 (1.18; 1.26) | 1.58 (1.51; 1.66) | 1.20 (1.16; 1.24) |
|  | Imidazoline receptor agonists | 1.33 (0.85; 2.06) | 1.41 (1.07; 1.86) | 1.17 (0.75; 1.81) | 1.14 (0.86; 1.50) | 0.91 (0.59; 1.42) | 1.04 (0.79; 1.38) |
|  | Alpha-adrenoreceptor antagonists | 1.42 (1.22; 1.66) | 1.21 (1.08; 1.36) | 1.36 (1.17; 1.59) | 1.11 (0.99; 1.25) | 1.15 (0.98; 1.34) | 1.05 (0.93; 1.19) |
|  |  |  |  |  |  |  |  |
| Anxiety disorders | Calcium Channel Blocker (CCB) | 1.28 (1.24; 1.32) | 1.06 (1.03; 1.09) | 1.28 (1.23; 1.32) | 1.03 (1.00; 1.06) | 1.19 (1.15; 1.24) | 1.01 (0.98; 1.04) |
|  | Diuretics | 1.16 (1.12; 1.21) | 1.04 (1.02; 1.07) | 1.16 (1.12; 1.21) | 1.00 (0.98; 1.03) | 1.01 (0.97; 1.05) | 0.98 (0.95; 1.01) |
|  | Angiotensin-Converting Enzyme inhibitors (ACE inhibitors) | 1.07 (1.04; 1.11) | 0.95 (0.93; 0.98) | 1.09 (1.05; 1.13) | 0.95 (0.93; 0.98) | 1.05 (1.01; 1.09) | 0.96 (0.94; 0.99) |
|  | Angiotensin II Receptor Blockers (ARB) | 1.24 (1.20; 1.29) | 1.08 (1.05; 1.11) | 1.24 (1.20; 1.29) | 1.04 (1.01; 1.07) | 1.18 (1.14; 1.23) | 1.04 (1.01; 1.07) |
|  | Beta blockers (BBs) | 1.76 (1.70; 1.83) | 1.27 (1.24, 1.31) | 1.71 (1.65; 1.78) | 1.18 (1.15, 1.21) | 1.64 (1.58; 1.71) | 1.19 (1.16, 1.22) |
|  | Imidazoline receptor agonists | 1.49 (1.06; 2.10) | 1.42 (1.12; 1.79) | 1.37 (0.98; 1.94) | 1.23 (0.97; 1.56) | 1.14 (0.81; 1.60) | 1.22 (0.96; 1.55) |
|  | Alpha-adrenoreceptor antagonists | 1.29 (1.13; 1.47) | 0.99 (0.89; 1.11) | 1.26 (1.11; 1.44) | 0.94 (0.85; 1.05) | 1.07 (0.94; 1.22) | 0.92 (0.82; 1.02) |
|  |  |  |  |  |  |  |  |
| Sleep disorders | Calcium Channel Blocker (CCB) | 1.48 (1.43; 1.52) | 1.23 (1.20; 1.26) | 1.47 (1.43; 1.51) | 1.19 (1.16; 1.22) | 1.33 (1.29; 1.37) | 1.13 (1.10; 1.16) |
|  | Diuretics | 1.40 (1.36; 1.45) | 1.26 (1.24; 1.29) | 1.40 (1.35; 1.44) | 1.21 (1.19; 1.24) | 1.18 (1.14; 1.22) | 1.13 (1.10; 1.16) |
|  | Angiotensin-Converting Enzyme inhibitors (ACE inhibitors) | 1.23 (1.20; 1.27) | 1.03 (1.00; 1.05) | 1.25 (1.21; 1.30) | 1.01 (0.99; 1.04) | 1.22 (1.18; 1.26) | 1.01 (0.99; 1.04) |
|  | Angiotensin II Receptor Blockers (ARB) | 1.38 (1.33; 1.42) | 1.24 (1.22; 1.27) | 1.38 (1.34; 1.42) | 1.19 (1.17; 1.22) | 1.29 (1.24; 1.33) | 1.16 (1.13; 1.19) |
|  | Beta blockers (BBs) | 1.58 (1.53; 1.64) | 1.27 (1.24; 1.29) | 1.54 (1.49; 1.59) | 1.18 (1.16; 1.21) | 1.39 (1.34; 1.44) | 1.14 (1.12; 1.17) |
|  | Imidazoline receptor agonists | 2.18 (1.68; 2.81) | 1.24 (1.01; 1.53) | 2.01 (1.56; 2.60) | 1.09 (0.88; 1.34) | 1.42 (1.09; 1.84) | 0.94 (0.77; 1.16) |
|  | Alpha-adrenoreceptor antagonists | 1.70 (1.54; 1.88) | 1.24 (1.14; 1.34) | 1.65 (1.49; 1.82) | 1.16 (1.07; 1.26) | 1.30 (1.17; 1.44) | 1.06 (0.98; 1.15) |
| Model A: Adjusting for Year of birth, Age at Hypertension, Education, Type of neighborhood, Country of birth. Model B: Adjusting for Year of birth, Age at Hypertension, Education, Type of neighborhood, Country of birth, Diabetes mellitus, Thyroid disorders, Opioids, Neuroleptics, Anxiolytics and sleep aids, Antidepressive, Metformin, Statins. Model C: Adjusted for Year of birth, Age at Hypertension, Education, Type of neighborhood, Country of birth, Diabetes mellitus, Thyroid disorders, Opioids, Neuroleptics, Anxiolytics and sleep aids, Antidepressive, Metformin, and Statins. All hypertensive drugs were included in the same model. | | | | | | | |

| **Table S11. Cox regression models with time to outcome and AHT as a time-dependent variable in both females and males** | | | | | | | |
| --- | --- | --- | --- | --- | --- | --- | --- |
| MALES AND FEMALES |  | MODEL A | | MODEL B | | MODEL C | |
|  |  | 0-2 years | 2 + years | 0-2 years | 2 + years | 0-2 years | 2 + years |
| OUTCOME | PREDICTOR | HR 95 % CI | HR 95 % CI | HR 95 % CI | HR 95 % CI | HR 95 % CI | HR 95 % CI |
| Substance abuse disorders | Calcium Channel Blocker (CCB) | 1.45 (1.40; 1.49) | 1.21 (1.18; 1.24) | 1.44 (1.40; 1.49) | 1.18 (1.15; 1.21) | 1.34 (1.30; 1.38) | 1.15 (1.12; 1.18) |
|  | Diuretics | 1.29 (1.25; 1.33) | 1.17 (1.14; 1.20) | 1.29 (1.24; 1.33) | 1.13 (1.10; 1.16) | 1.13 (1.09; 1.17) | 1.10 (1.08; 1.13) |
|  | Angiotensin-Converting Enzyme inhibitors (ACE inhibitors) | 1.31 (1.27; 1.35) | 1.11 (1.08; 1.13) | 1.32 (1.28; 1.36) | 1.09 (1.06; 1.11) | 1.23 (1.19; 1.27) | 1.05 (1.03; 1.08) |
|  | Angiotensin II Receptor Blockers (ARB) | 1.11 (1.07; 1.15 | 0.96 (0.94; 0.99) | 1.11 (1.07; 1.15) | 0.93 (0.91; 0.95) | 1.04 (1.00; 1.08) | 0.92 (0.89; 0.94) |
|  | Beta blockers (BBs) | 1.61 (1.55; 1.66) | 1.31 (1.28; 1.34) | 1.59 (1.54; 1.65) | 1.25 (1.22; 1.28) | 1.48 (1.43; 1.54) | 1.23 (1.20; 1.26) |
|  | Imidazoline receptor agonists | 1.81 (1.34; 2.43) | 1.09 (0.86; 1.38) | 1.70 (1.27; 2.29) | 0.98 (0.78; 1.24) | 1.48 (1.10; 1.99) | 0.98 (0.77; 1.24) |
|  | Alpha-adrenoreceptor antagonists | 0.92 (0.79; 1.08) | 0.83 (0.74; 0.93) | 0.89 (0.76; 1.05) | 0.79 (0.71; 0.89) | 0.72 (0.61; 0.84) | 0.74 (0.65; 0.83) |
|  |  |  |  |  |  |  |  |
| Psychotic disorders | Calcium Channel Blocker (CCB) | 1.05 (0.91; 1.21) | 1.00 (0.90; 1.12) | 1.05 (0.91; 1.21) | 0.98 (0.87; 1.09) | 1.04 (0.91; 1.20) | 1.00 (0.90; 1.12) |
|  | Diuretics | 1.02 (0.89; 1.17) | 0.95 (0.86; 1.05) | 1.02 (0.88; 1.17) | 0.91 (0.82; 1.00) | 1.00 (0.86; 1.15) | 0.94 (0.85; 1.04) |
|  | Angiotensin-Converting Enzyme inhibitors (ACE inhibitors) | 0.98 (0.85; 1.14) | 0.98 (0.88; 1.08) | 0.98 (0.85; 1.13) | 0.93 (0.85; 1.03) | 0.92 (0.80; 1.07) | 0.92 (0.84; 1.02) |
|  | Angiotensin II Receptor Blockers (ARB) | 0.79 (0.67; 0.93) | 0.84 (0.76; 0.94) | 0.79 (0.67; 0.93) | 0.81 (0.73; 0.90) | 0.76 (0.64; 0.90) | 0.82 (0.73; 0.91) |
|  | Beta blockers (BBs) | 1.48 (1.29; 1.69) | 1.21 (1.10; 1.34) | 1.46 (1.27; 1.67) | 1.15 (1.04; 1.27) | 1.46 (1.27; 1.68) | 1.17 (1.06; 1.29) |
|  | Imidazoline receptor agonists | 1.30 (0.32; 5.21) | 1.20 (0.50; 2.88) | 1.16 (0.29; 4.65) | 1.00 (0.41; 2.42) | 1.07 (0.26; 4.43) | 1.13 (0.46; 2.75) |
|  | Alpha-adrenoreceptor antagonists | 1.63 (0.98; 2.71) | 0.73 (0.43; 1.24) | 1.52 (0.92; 2.53) | 0.68 (0.40; 1.16) | 1.51 (0.90; 2.54) | 0.70 (0.41; 1.19) |
|  |  |  |  |  |  |  |  |
| Affective disorders | Calcium Channel Blocker (CCB) | 1.24 (1.21; 1.27) | 1.12 (1.09; 1.14) | 1.23 (1.20; 1.26) | 1.07 (1.05; 1.09) | 1.17 (1.14; 1.20) | 1.05 (1.02; 1.07) |
|  | Diuretics | 1.20 (1.17; 1.24) | 1.18 (1.15; 1.20) | 1.19 (1.16; 1.22) | 1.09 (1.07; 1.11) | 1.09 (1.06; 1.12) | 1.06 (1.04; 1.08) |
|  | Angiotensin-Converting Enzyme inhibitors (ACE inhibitors) | 1.07 (1.04; 1.10) | 1.02 (1.00; 1.04) | 1.09 (1.06; 1.12) | 0.99 (0.97; 1.01) | 1.04 (1.02; 1.07) | 0.99 (0.97; 1.01) |
|  | Angiotensin II Receptor Blockers (ARB) | 1.16 (1.13; 1.19) | 1.13 (1.10; 1.15) | 1.16 (1.13; 1.19) | 1.06 (1.04; 1.08) | 1.10 (1.07; 1.13) | 1.04 (1.02; 1.06) |
|  | Beta blockers (BBs) | 1.44 (1.40; 1.48) | 1.21 (1.19; 1.24) | 1.41 (1.37; 1.45) | 1.10 (1.08; 1.13) | 1.35 (1.32; 1.39) | 1.09 (1.07; 1.11) |
|  | Imidazoline receptor agonists | 1.57 (1.21; 2.02) | 1.46 (1.23; 1.72) | 1.39 (1.08; 1.80) | 1.19 (1.01; 1.40) | 1.16 (0.89; 1.50) | 1.12 (0.94; 1.32) |
|  | Alpha-adrenoreceptor antagonists | 1.39 (1.24; 1.56) | 1.21 (1.11; 1.33) | 1.33 (1.18; 1.48) | 1.13 (1.03; 1.23) | 1.16 (1.03; 1.30) | 1.08 (0.98; 1.18) |
|  |  |  |  |  |  |  |  |
| Bipolar disorders | Calcium Channel Blocker (CCB) | 0.93 (0.77; 1.13) | 0.94 (0.82; 1.09) | 0.92 (0.76; 1.12) | 0.89 (0.77; 1.03) | 0.90 (0.74; 1.09) | 0.92 (0.80; 1.07) |
|  | Diuretics | 1.22 (1.02; 1.45) | 1.07 (0.94; 1.22) | 1.20 (1.01; 1.43) | 0.99 (0.87; 1.12) | 1.18 (0.98; 1.41) | 1.04 (0.91; 1.18) |
|  | Angiotensin-Converting Enzyme inhibitors (ACE inhibitors) | 0.98 (0.81; 1.18) | 0.85 (0.75; 0.97) | 0.99 (0.82; 1.19) | 0.82 (0.72; 0.93) | 0.94 (0.78; 1.14) | 0.82 (0.72; 0.94) |
|  | Angiotensin II Receptor Blockers (ARB) | 0.98 (0.80; 1.20) | 1.00 (0.88; 1.15) | 0.98 (0.80; 1.20) | 0.94 (0.82; 1.08) | 0.93 (0.76; 1.13) | 0.93 (0.80; 1.07) |
|  | Beta blockers (BBs) | 1.42 (1.19; 1.70) | 1.17 (1.03; 1.33) | 1.38 (1.15; 1.64) | 1.04 (0.92; 1.18) | 1.34 (1.12; 1.61) | 1.05 (0.92; 1.19) |
|  | Imidazoline receptor agonists | 1.15 (0.16; 8.16) | 1.39 (0.45; 4.32) | 0.94 (0.13; 6.70) | 1.00 (0.32; 3.12) | 0.98 (0.14; 6.63) | 1.04 (0.33; 3.25) |
|  | Alpha-adrenoreceptor antagonists | 0.79 (0.30; 2.09) | 1.12 (0.62; 2.03) | 0.72 (0.27; 1.92) | 1.01 (0.56; 1.83) | 0.71 (0.27; 1.86) | 1.06 (0.58; 1.93) |
|  |  |  |  |  |  |  |  |
| Depressive disorders | Calcium Channel Blocker (CCB) | 1.25 (1.22; 1.28) | 1.12 (1.09; 1.14) | 1.24 (1.21; 1.28) | 1.07 (1.05; 1.09) | 1.18 (1.14; 1.21) | 1.04 (1.02; 1.07) |
|  | Diuretics | 1.21 (1.18; 1.24) | 1.18 (1.16; 1.20) | 1.20 (1.17; 1.23) | 1.09 (1.07; 1.11) | 1.09 (1.06; 1.12) | 1.07 (1.05; 1.09) |
|  | Angiotensin-Converting Enzyme inhibitors (ACE inhibitors) | 1.08 (1.05; 1.11) | 1.03 (1.01; 1.05) | 1.10 (1.07; 1.13) | 1.00 (0.98; 1.02) | 1.05 (1.02; 1.08) | 0.99 (0.97; 1.01) |
|  | Angiotensin II Receptor Blockers (ARB) | 1.16 (1.13; 1.20) | 1.13 (1.10; 1.15) | 1.16 (1.13; 1.20) | 1.06 (1.04; 1.08) | 1.10 (1.07; 1.14) | 1.04 (1.02; 1.06) |
|  | Beta blockers (BBs) | 1.45 (1.41; 1.49) | 1.23 (1.20; 1.25) | 1.42 (1.38; 1.46) | 1.12 (1.09; 1.14) | 1.36 (1.32; 1.40) | 1.10 (1.08; 1.12) |
|  | Imidazoline receptor agonists | 1.60 (1.24; 2.07) | 1.45 (1.23; 1.72) | 1.44 (1.11; 1.86) | 1.19 (1.01; 1.41) | 1.19 (0.92; 1.54) | 1.12 (0.95; 1.33) |
|  | Alpha-adrenoreceptor antagonists | 1.42 (1.27; 1.59) | 1.21 (1.11; 1.33) | 1.35 (1.21; 1.52) | 1.13 (1.03; 1.23) | 1.18 (1.05; 1.32) | 1.07 (0.98; 1.17) |
|  |  |  |  |  |  |  |  |
| Anxiety disorders | Calcium Channel Blocker (CCB) | 1.29 (1.26; 1.31) | 1.06 (1.04; 1.08) | 1.28 (1.25; 1.31) | 1.03 (1.01; 1.05) | 1.21 (1.19; 1.24) | 1.02 (1.00; 1.03) |
|  | Diuretics | 1.14 (1.11; 1.16) | 1.06 (1.05; 1.08) | 1.13 (1.10; 1.15) | 1.00 (0.99; 1.02) | 1.00 (0.98; 1.03) | 0.99 (0.97; 1.00) |
|  | Angiotensin-Converting Enzyme inhibitors (ACE inhibitors) | 1.09 (1.07; 1.11) | 0.96 (0.95; 0.97) | 1.11 (1.08; 1.13) | 0.95 (0.94; 0.97) | 1.07 (1.04; 1.09) | 0.97 (0.95; 0.98) |
|  | Angiotensin II Receptor Blockers (ARB) | 1.20 (1.18; 1.23) | 1.1 0(1.08; 1.11) | 1.21 (1.18; 1.23) | 1.06 (1.04; 1.07) | 1.15 (1.13; 1.18) | 1.06 (1.04; 1.08) |
|  | Beta blockers (BBs) | 1.60 (1.57; 1.63) | 1.21 (1.19; 1.23) | 1.57 (1.53; 1.60) | 1.13 (1.12; 1.15) | 1.51 (1.48; 1.54) | 1.13 (1.12; 1.15) |
|  | Imidazoline receptor agonists | 1.57 (1.27; 1.94) | 1.49 (1.30; 1.70) | 1.44 (1.16; 1.78) | 1.29 (1.12; 1.48) | 1.22 (0.98; 1.51) | 1.27 (1.10; 1.46) |
|  | Alpha-adrenoreceptor antagonists | 1.29 (1.17; 1.42) | 1.02 (0.95; 1.11) | 1.27 (1.15; 1.39) | 0.98 (0.91; 1.06) | 1.08 (0.98; 1.19) | 0.94 (0.87; 1.02) |
|  |  |  |  |  |  |  |  |
| Sleep disorders | Calcium Channel Blocker (CCB) | 1.35 (1.32; 1.38) | 1.15 (1.13; 1.16) | 1.34 (1.32; 1.37) | 1.10 (1.09; 1.12) | 1.25 (1.23; 1.28) | 1.06 (1.04; 1.08) |
|  | Diuretics | 1.27 (1.24; 1.30) | 1.24 (1.22; 1.26) | 1.26 (1.24; 1.29) | 1.17 (1.15; 1.19) | 1.12 (1.10; 1.15) | 1.12 (1.10; 1.14) |
|  | Angiotensin-Converting Enzyme inhibitors (ACE inhibitors) | 1.16 (1.13; 1.18) | 1.00 (0.98; 1.01) | 1.18 (1.15; 1.20) | 0.99 (0.97; 1.00) | 1.14 (1.12; 1.17) | 0.98 (0.97; 1.00) |
|  | Angiotensin II Receptor Blockers (ARB) | 1.31 (1.28; 1.34) | 1.22 (1.21; 1.24) | 1.31 (1.29; 1.34) | 1.17 (1.15; 1.19) | 1.24 (1.21; 1.27) | 1.14 (1.12; 1.15) |
|  | Beta blockers (BBs) | 1.40 (1.37; 1.43) | 1.23 (1.22; 1.25) | 1.36 (1.33; 1.39) | 1.14 (1.12; 1.15) | 1.27 (1.24; 1.30) | 1.12 (1.10; 1.13) |
|  | Imidazoline receptor agonists | 2.15 (1.80; 2.57) | 1.35 (1.18; 1.54) | 1.98 (1.65; 2.37) | 1.15 (1.01; 1.32) | 1.54 (1.29; 1.85) | 1.05 (0.92; 1.21) |
|  | Alpha-adrenoreceptor antagonists | 1.55 (1.43; 1.68) | 1.15 (1.08; 1.23) | 1.49 (1.37; 1.62) | 1.08 (1.01; 1.16) | 1.23 (1.13; 1.34) | 1.00 (0.94; 1.07) |
| Model A: Adjusting for Year of birth, Age at Hypertension, Education, Type of neighborhood, Country of birth. Model B: Adjusting for Year of birth, Age at Hypertension, Education, Type of neighborhood, Country of birth, Diabetes mellitus, Thyroid disorders, Opioids, Neuroleptics, Anxiolytics and sleep aids, Antidepressive, Metformin, Statins. Model C: Adjusted for Year of birth, Age at Hypertension, Education, Type of neighborhood, Country of birth, Diabetes mellitus, Thyroid disorders, Opioids, Neuroleptics, Anxiolytics and sleep aids, Antidepressive, Metformin, and Statins. All hypertensive drugs were included in the same model. | | | | | | | |

| **Table S12. Co-sibling analysis** | | | |
| --- | --- | --- | --- |
| MALES AND FEMALES |  | Sibling Model | |
|  |  | 0-2 years | 2 + years |
| OUTCOME | PREDICTOR | HR 95 % CI | HR 95 % CI |
| Substance abuse disorders | Calcium Channel Blocker (CCB) | 1.43 (1.22; 1.69) | 1.02 (0.87; 1.21) |
|  | Diuretics | 1.08 (0.92; 1.27) | 1.02 (0.86; 1.21) |
|  | Angiotensin-Converting Enzyme inhibitors (ACE inhibitors) | 1.40 (1.13; 1.74) | 1.14 (0.92; 1.42) |
|  | Angiotensin II Receptor Blockers (ARB) | 1.58 (1.28; 1.96) | 1.16 (0.92; 1.47) |
|  | Beta blockers (BBs) | 1.55 (1.26; 1.91) | 1.32 (1.07; 1.64) |
|  | Imidazoline receptor agonists | No Info | No Info |
|  | Alpha-adrenoreceptor antagonists | No Info | No Info |
|  |  |  |  |
| Psychotic disorders | Calcium Channel Blocker (CCB) | 0.99 (0.41; 2.38) | 0.90 (0.36; 2.27) |
|  | Diuretics | 0.38 (0.18; 0.78) | 0.47 (0.13; 1.67) |
|  | Angiotensin-Converting Enzyme inhibitors (ACE inhibitors) | 47.4 (5.17; 44.4) | 1.56 (0.30; 8.21) |
|  | Angiotensin II Receptor Blockers (ARB) | 0.60 (0.20; 1.81) | 1.37 (0.29; 6.54) |
|  | Beta blockers (BBs) | 1.99 (0.63; 6.21) | 3.05 (0.74; 12.6) |
|  | Imidazoline receptor agonists | No Info | No Info |
|  | Alpha-adrenoreceptor antagonists | No Info | No Info |
|  |  |  |  |
| Affective disorders | Calcium Channel Blocker (CCB) | 1.41 (1.22; 1.62) | 0.92 (0.79; 1.08) |
|  | Diuretics | 1.19 (1.03; 1.36) | 1.04 (0.89; 1.22) |
|  | Angiotensin-Converting Enzyme inhibitors (ACE inhibitors) | 1.47 (1.23; 1.77) | 1.05 (0.86; 1.28) |
|  | Angiotensin II Receptor Blockers (ARB) | 1.73 (1.46; 2.05) | 1.03 (0.87; 1.23) |
|  | Beta blockers (BBs) | 1.47 (1.23; 1.75) | 1.09 (0.91; 1.30) |
|  | Imidazoline receptor agonists | No Info | No Info |
|  | Alpha-adrenoreceptor antagonists | No Info | No Info |
|  |  |  |  |
| Bipolar disorders | Calcium Channel Blocker (CCB) | 0.92 (0.33; 2.56) | 0.41 (0.13; 1.31) |
|  | Diuretics | 7.15 (1.20; 42.6) | 0.54 (0.07; 4.28) |
|  | Angiotensin-Converting Enzyme inhibitors (ACE inhibitors) | 9.77 (0.75; 127) | 1.63 (0.18; 15.2) |
|  | Angiotensin II Receptor Blockers (ARB) | 35.4 (4.80; 262) | 6.33 (0.62; 64.9) |
|  | Beta blockers (BBs) | 3.52 (0.49; 25.4) | 1.30 (0.24; 7.12) |
|  | Imidazoline receptor agonists | No Info | No Info |
|  | Alpha-adrenoreceptor antagonists | No Info | No Info |
|  |  |  |  |
| Depressive disorders | Calcium Channel Blocker (CCB) | 1.42 (1.23; 1.64) | 0.92 (0.79; 1.08) |
|  | Diuretics | 1.16 (1.01; 1.33) | 1.03 (0.88; 1.21) |
|  | Angiotensin-Converting Enzyme inhibitors (ACE inhibitors) | 1.41 (1.18; 1.70) | 1.01 (0.82; 1.24) |
|  | Angiotensin II Receptor Blockers (ARB) | 1.74 (1.46; 2.06) | 1.05 (0.88; 1.25) |
|  | Beta blockers (BBs) | 1.41 (1.17; 1.68) | 1.07 (0.90; 1.28) |
|  | Imidazoline receptor agonists | No Info | No Info |
|  | Alpha-adrenoreceptor antagonists | No Info | No Info |
|  |  |  |  |
| Anxiety disorders | Calcium Channel Blocker (CCB) | 1.48 (1.31; 1.66) | 0.99 (0.87; 1.13) |
|  | Diuretics | 1.23 (1.09; 1.38) | 1.00 (0.87; 1.15) |
|  | Angiotensin-Converting Enzyme inhibitors (ACE inhibitors) | 1.01 (0.86; 1.19) | 1.06 (0.89; 1.26) |
|  | Angiotensin II Receptor Blockers (ARB) | 1.36 (1.19; 1.56) | 1.13 (0.97; 1.31) |
|  | Beta blockers (BBs) | 1.30 (1.12; 1.51) | 0.96 (0.82; 1.13) |
|  | Imidazoline receptor agonists | No Info | No Info |
|  | Alpha-adrenoreceptor antagonists | No Info | No Info |
|  |  |  |  |
| Sleep disorders | Calcium Channel Blocker (CCB) | 1.27 (1.14; 1.41) | 0.97 (0.87; 1.09) |
|  | Diuretics | 1.15 (1.03; 1.28) | 1.14 (1.02; 1.27) |
|  | Angiotensin-Converting Enzyme inhibitors (ACE inhibitors) | 1.27 (1.09; 1.46) | 1.10 (0.94; 1.27) |
|  | Angiotensin II Receptor Blockers (ARB) | 1.27 (1.13; 1.44) | 1.23 (1.09; 1.38) |
|  | Beta blockers (BBs) | 1.26 (1.09; 1.44) | 0.99 (0.86; 1.13) |
|  | Imidazoline receptor agonists | No Info | No Info |
|  | Alpha-adrenoreceptor antagonists | No Info | No Info |
| Adjusting for Year of birth, Age at Hypertension, Education, Type of neighborhood, Country of birth. | | | |
